# Blockade of TGF-**β** signaling reactivates HIV-1/SIV reservoirs and immune responses in vivo

**DOI:** 10.1172/jci.insight.162290

**Published:** 2022-11-08

**Authors:** Sadia Samer, Yanique Thomas, Mariluz Araínga, Crystal Carter, Lisa M. Shirreff, Muhammad S. Arif, Juan M. Avita, Ines Frank, Michael D. McRaven, Christopher T. Thuruthiyil, Veli B. Heybeli, Meegan R. Anderson, Benjamin Owen, Arsen Gaisin, Deepanwita Bose, Lacy M. Simons, Judd F. Hultquist, James Arthos, Claudia Cicala, Irini Sereti, Philip J. Santangelo, Ramon Lorenzo-Redondo, Thomas J. Hope, Francois J. Villinger, Elena Martinelli

**Affiliations:** 1 Department of Cell and Developmental Biology, Feinberg School of Medicine, Northwestern University, Chicago, Illinois, USA.; 2 New Iberia Research Center (NIRC), University of Louisiana at Lafayette, New Iberia, Louisiana, USA.; 3 Center for Biomedical Research, Population Council, New York, New York, USA.; 4 Integrated Molecular Structure Education and Research (IMSERC), Northwestern University, Evanston, Illinois, USA.; 5 Department of Medicine, Division of Infectious Diseases, Feinberg School of Medicine, Northwestern University, Chicago, Illinois, USA.; 6 Center for Pathogen Genomics and Microbial Evolution, Havey Institute for Global Health Northwestern University, Chicago, Illinois, USA.; 7 Laboratory of Immunoregulation, National Institute of Allergy and Infectious Diseases (NIAID), National Institutes of Health (NIH), Bethesda, Maryland, USA.; 8 WH Coulter Department of Biomedical Engineering, Emory University School of Medicine, Atlanta, Georgia, USA.

**Keywords:** AIDS/HIV, Cytokines, Immunotherapy, T cells

## Abstract

TGF-β plays a critical role in maintaining immune cells in a resting state by inhibiting cell activation and proliferation. Resting HIV-1 target cells represent the main cellular reservoir after long-term antiretroviral therapy (ART). We hypothesized that releasing cells from TGF-β–driven signaling would promote latency reversal. To test our hypothesis, we compared HIV-1 latency models with and without TGF-β and a TGF-β type 1 receptor inhibitor, galunisertib. We tested the effect of galunisertib in SIV-infected, ART-treated macaques by monitoring SIV-env expression via PET/CT using the ^64^Cu-DOTA-F(ab′)_2_ p7D3 probe, along with plasma and tissue viral loads (VLs). Exogenous TGF-β reduced HIV-1 reactivation in U1 and ACH-2 models. Galunisertib increased HIV-1 latency reversal ex vivo and in PBMCs from HIV-1–infected, ART-treated, aviremic donors. In vivo, oral galunisertib promoted increased total standardized uptake values in PET/CT images in gut and lymph nodes of 5 out of 7 aviremic, long-term ART-treated, SIV-infected macaques. This increase correlated with an increase in SIV RNA in the gut. Two of the 7 animals also exhibited increases in plasma VLs. Higher anti-SIV T cell responses and antibody titers were detected after galunisertib treatment. In summary, our data suggest that blocking TGF-β signaling simultaneously increases retroviral reactivation events and enhances anti-SIV immune responses.

## Graphical abstract

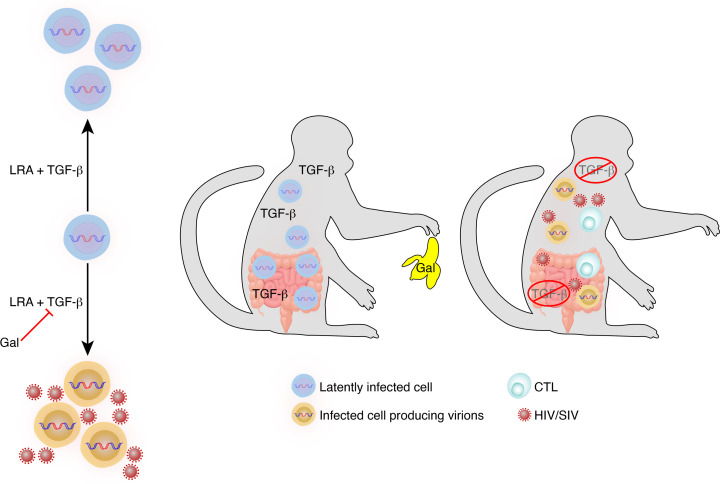

## Introduction

Despite the success of combination antiretroviral therapy (ART) in suppressing viral replication and preventing immunodeficiency progression, HIV-1 infection persists and evades immune responses in viral reservoirs, which are considered the major obstacle to HIV-1 cure (1). Long-lived CD4^+^ T cells that expand by homeostatic or antigen-driven proliferation in blood and tissues constitute a reservoir of proviruses that may be fully latent or transcriptionally active to a different extent in different CD4^+^ T cells (2, 3). Myeloid cells may also constitute an important cellular reservoir (4), though HIV-1 latency in the myeloid compartment has only recently been explored (5, 6). Viral rebound upon ART interruption occurs in the vast majority of patients, likely following stochastic HIV-1 reactivation events, and its dynamics depend on the size of the reservoir and host immune factors (7–9). “Shock and kill” strategies have the potential to lead to a functional HIV-1 cure by reducing reservoir size and stimulating immune responses (10, 11). However, the success of this strategy is hindered by several obstacles, including the vast heterogeneity of the cells comprising the reservoir and of the mechanisms of latency (2, 12) and the low inducibility of the latent proviruses (13, 14).

TGF-β is one of the most potent endogenous immunosuppressive factors, and it is considered the master regulator of mucosal immunity (15, 16). TGF-β signaling regulates diverse cellular processes, including proliferation, differentiation, and migration (17), and influences developmental as well as pathological processes such as epithelial-mesenchymal transition in fibrosis (18). In the immune system, TGF-β is essential to the maintenance of tolerance (19, 20). Importantly, TGF-β is the most prominent factor responsible for the maintenance of a resting state in CD4^+^ T cells (21), and loss of TGF-β responsiveness in mature T cells decreases their threshold for activation (22). Moreover, TGF-β also affects activation status and function of myeloid cells by decreasing antigen presentation capability and maturation and inducing tolerogenic properties (23, 24).

Since the beginning of the HIV-1 epidemic, several reports have documented higher concentrations of TGF-β in the blood, lymphoid tissues, and cerebrospinal fluid of people living with HIV-1 (PLWH) (25, 26). Higher levels of TGF-β in PLWH and SIV-infected macaques have been linked to disease progression (25, 27, 28). PBMCs from PLWH spontaneously release high levels of TGF-β (29, 30). Among other mechanisms, TGF-β release particularly in the gut tissue is the result of a biological antiinflammatory process counteracting HIV-driven microbial translocation and chronic inflammation (31). Paradoxically, this antiinflammatory, TGF-β–centered response not only exacerbates immunosuppression but also predisposes for the development of non–AIDS-related, noncommunicable disorders (31). Finally, TGF-β–driven fibrosis of lymphoid tissues has been implicated in the CD4^+^ T cell loss before ART initiation (32) and failure to achieve full immune reconstitution with ART (33).

Importantly, TGF-β inhibits TCR-induced proliferation of, CD28-mediated costimulation of, and activation of T cells (34, 35). Indeed, it has recently been used to promote latency in ex vivo models with primary polarized effector CD4^+^ T cells (36, 37). Moreover, TGF-β was shown to induce latency in other cell types susceptible to HIV infection (38).

The 3 isoforms of TGF-β (TGF-β1, TGF-β2, and TGF-β3) bind to the specific type 1 receptor TGFBR1/ALK5, which then undergoes dimerization with the type 2 TGF-βRII. The differential function and signaling mechanism of these major isoforms are still under investigation. However, TGF-β1 appears to be the most prominent regulator of immune responses (39, 40). The TGF-β receptor complex then signals through a canonical pathway by phosphorylating Smad2 and/or Smad3. The Smad complex, formed by coheterodimers with Smad4, interacts with a large variety of coactivators and corepressors in the nucleus and regulates the transcription of hundreds of genes in a cell type–specific, context-dependent way (20). Galunisertib (LY2157299), a small-molecule selective inhibitor of the TGFBR1/ALK5 (41, 42), has been developed by Eli Lilly as an anticancer therapeutic to target the direct and indirect effect of TGF-β on tumor growth. Galunisertib progressed to an advanced stage of clinical development (phase II) and had been tested in several different trials for different solid tumors (42–44), when Eli Lilly decided to end its program to pursue more promising targets (45).

Given the suppressive activity of TGF-β on different immune cell targets of HIV-1 infection and its role in latency induction (36, 38), we hypothesized that TGF-β may also exert a suppressive effect on latency reactivation. Moreover, we hypothesized that if TGF-β inhibited TCR- or PKC-driven HIV reactivation from latency, inhibition of TGF-β signaling may, in turn, increase the frequency of latency reversal following stimulation of these pathways. We tested this hypothesis using ex vivo models of HIV latency including PBMCs from aviremic, ART-treated PLWH. Finally, we tested the impact of the TGFBR1 inhibitor galunisertib ex vivo and in SIV-infected ART-treated macaques. Our data suggest that targeting TGF-β signaling may constitute a way to increase viral latency reactivation during ART while potentiating antiviral immune responses.

## Results

### TGF-β1 inhibits PMA-driven and DC-driven HIV-1 latency reactivation in vitro.

**Figure 1:**
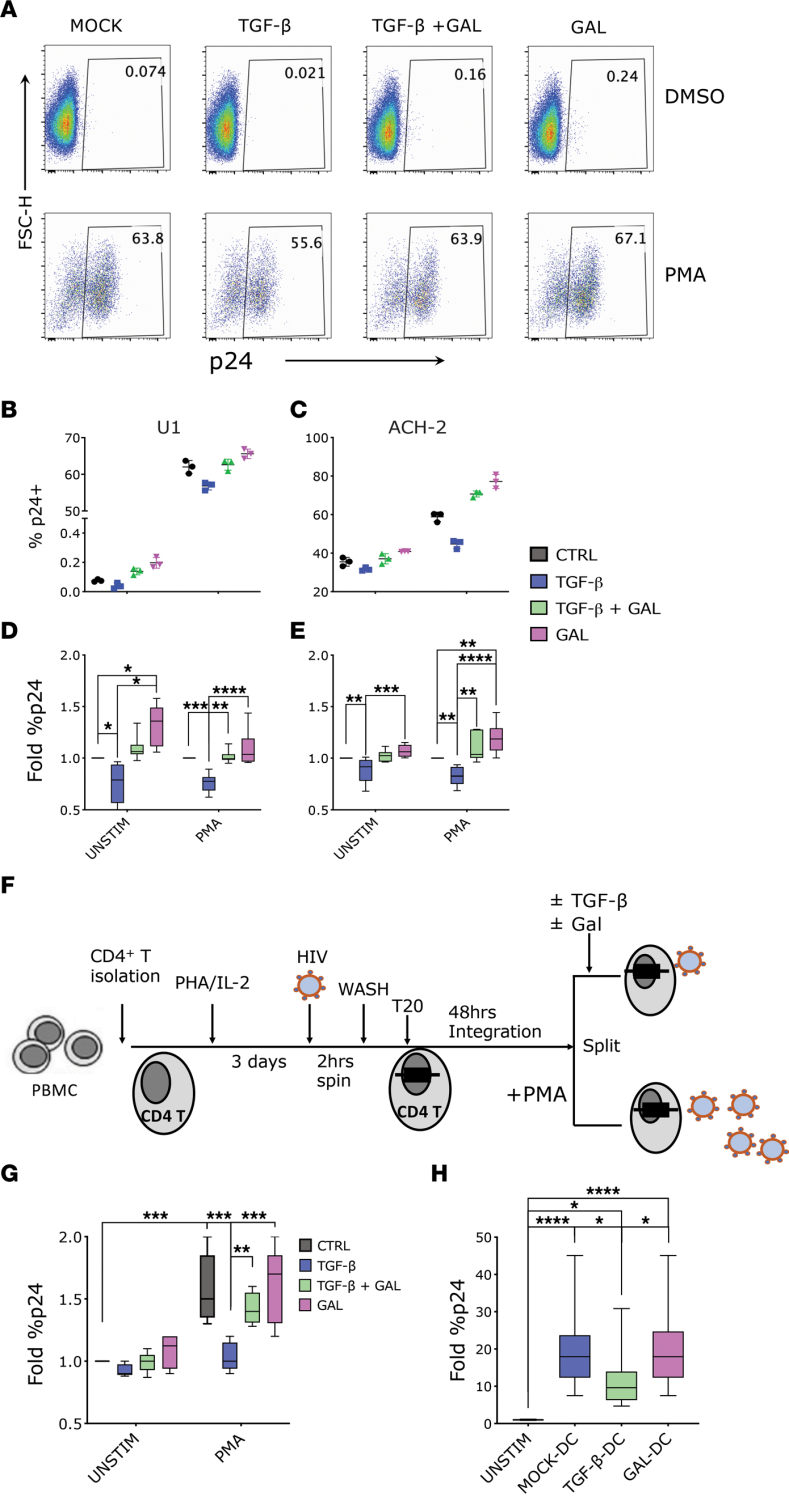
TGF-β inhibits HIV-1 latency reactivation in vitro. (**A**–**E**) U1 and ACH-2 cells were treated with TGF-β1 (10 ng/mL) or galunisertib (GAL) (1 μM) or both or were mock treated in presence versus absence of PMA (100 ng/mL) for 18 hours and stained for intracellular p24. (**A**) An example of p24 detection in 1 experiment with U1 cells. (**B** and **C**) Raw p24 data from 1 representative experiment with U1 (**B**) or ACH-2 (**C**) cells (black circles: mock; blue squares: TGF-β1; green triangles: TGF-β1 + gal; pink triangles: galunisertib only). (**D** and **E**) Summary fold increase in the frequency of p24^+^ cells over the mock condition (box plot with median line and min/max whiskers) shown from 5 similar experiments (U1 on the left; ACH-2 on the right). (**F** and **G**) For this primary CD4^+^ T cell model of latency, CD4^+^ T cells were isolated from PBMCs, activated, infected by spinoculation, and incubated for 2 days in the presence of T20. PMA (100 ng/mL) was used for reactivation of latently infected cells for 18 hours in the presence of TGF-β1 (10 ng/mL) or galunisertib (1 μM), both, or mock treatment. A schematic of the experiment is shown in **F**. (**G**) Summary data (fold increase over the unstimulated condition) from 5 experiments with cells from different donors run in triplicate. (**H**) Data from a model of DC-driven HIV reactivation from U1 cells are shown. Fold difference in the frequency of p24^+^ U1 cells in absence versus presence of moDCs or TGF-β DCs shown for 5 different experiments in triplicate (box plot with median and min/max whiskers). (**A**–**H**) Conditions were compared by Kruskal-Wallis ANOVA test followed by the Dunn’s test corrected for multiple comparisons. Significant *P* values of α < 0.05 (*), α < 0.01 (**), and α < 0.001 (***) are indicated. All other comparisons were nonsignificant. Box-and-whisker plots represent median, 25th and 75th percentile, with whiskers going from min to max.

We tested the impact of exogenous TGF-β1 on PMA-induced HIV reactivation in the U1 and ACH-2 classical cell line models of HIV latency (U1 derived from U937 promonocytic cells, ref. 46, and ACH-2 derived from CEM T cells, ref. 47). U1 cells originally contained 2 copies of integrated HIV DNA, although more recently HIV was detected in additional sites (48), and latency is maintained by mutations in the Tat protein (46). The frequency of U1 cells expressing p24-gag at baseline was very low (Figure 1A, MOCK-DMSO), but it was readily induced by stimulation with PMA (Figure 1A, MOCK-PMA). Addition of 10 ng/mL of TGF-β1 to the culturing media decreased the frequency of p24-gag^+^ cells below the baseline level in unstimulated cultures and reduced the PMA-driven increase in the frequency of p24-gag^+^ cells by approximately 20% (Figure 1, A, B, and D). In contrast, blockade of TGF-β1 signaling using galunisertib restored the frequency of p24-gag^+^ cells in the presence of exogenous TGF-β1 to baseline or PMA-induced levels. Treatment with 1 μM of galunisertib alone appeared to slightly increase the frequency of p24-gag^+^ cells from baseline but had no impact on PMA-stimulated U1 cells (Figure 1D).

ACH-2 cells were originally reported to carry a single copy of HIV DNA (47), but, as with U1, HIV integration has been detected in multiple sites in more recent culture passages (48). Latency is maintained by a mutation in the HIV trans-activation response element that impairs the long terminal repeat response to Tat (49). In contrast to U1, the frequency of p24-gag^+^ cells was relatively high in ACH-2 at baseline (Supplemental Figure 1; supplemental material available online with this article; https://doi.org/10.1172/jci.insight.162290DS1; MOCK-DMSO) but increased further in response to PMA (Supplemental Figure 1, MOCK-PMA). We found that TGF-β1 was able to decrease the frequency of p24-gag^+^ cells in PMA-stimulated cultures by approximately 20%, and a smaller but consistent decrease in baseline p24-gag levels was also detected in the absence of PMA (Figure 1, C and E). Galunisertib treatment was able to restore frequency of p24-gag^+^ cells in TGF-β1–treated cultures and increase it above PMA-stimulated cultures (Figure 1, C and E) when administered in absence of exogenous TGF-β1. This increase may be attributed to blockade of TGF-β1 in the fetal bovine serum (FBS) used to supplement the culturing media (21). Indeed, in control experiments performed in X-Vivo 15 medium without FBS, this galunisertib-driven increase was absent, and the impact of TGF-β1 was more pronounced (Supplemental Figure 2). However, cell viability in X-Vivo 15 medium was significantly lower than in cultures with FBS for both U1 and ACH-2 cells (U1 and ACH-2 with FBS: means 98% and 96%, SD 3.4% and 2.4%; U1 and ACH-2 in X-Vivo: means 68% and 90.1%, SD 12.8% and 3.04%; *P* < 0.001 for both).

Next, we tested the impact of TGF-β1 on a primary model of HIV-1 latency using freshly isolated CD4^+^ T cells. The experiment was based on the model by Berkhout and colleagues (50) with some modifications (Figure 1F). Specifically, we isolated CD4^+^ T cells from HIV-1 uninfected, deidentified donor PBMCs and activated them with phytohemagglutinin (PHA) and IL-2 for 3 days before infection via spinoculation with HIV ADA. After extensive wash of the viral inoculum, cells were cultured 48 hours in the presence of T20 (enfuvirtide) to avoid viral spread beyond the cells with initial integrations. The overall initial infection achieved was very low despite the spinoculation step (~0.1% of p24-gag^+^ cells, Supplemental Figure 2). However, PMA stimulation was able to increase the frequency of p24-gag^+^ cells consistently (Figure 1G and Supplemental Figure 3), and this was significantly inhibited when TGF-β1 (10 ng/mL) was added concomitantly with PMA. In contrast, the addition of both TGF-β1 and galunisertib (1 μM) or galunisertib alone did not alter the frequency of p24-gag^+^ cells from the level in control cultures (Figure 1G and Supplemental Figure 3).

Finally, considering that DCs are important players in reversing HIV latency (51) and that TGF-β1 impairs DC activation (52, 53), we decided to test the impact of TGF-β1 on DC-mediated HIV-1 latency reactivation (50, 54). Monocyte-derived DCs (moDCs) were generated from primary monocytes as previously described (55) and treated or not with TGF-β1 for the last 2 days of culture. moDCs generated in the presence of TGF-β1 exhibited a less mature phenotype with tendency toward lower HLA-DR and significantly lower DEC-205 expression (Supplemental Figure 4A). When cocultured with eFluor670-prelabeled U1 cells, moDCs were able to promptly activate HIV-1 expression, with the frequency of p24-gag^+^ cells increasing of several folds within a few hours (Figure 1H and Supplemental Figure 3B). However, the frequency of p24-gag^+^ cells in the cocultures with TGF-β1–treated DCs was significantly less than in cocultures with untreated DCs, and this was blocked when TGF-β1 treatment of DCs occurred in the presence of galunisertib (Figure 1H and Supplemental Figure 4B).

### Blocking TGF-β1 signaling increases latency reversal agent–induced HIV-1 reactivation ex vivo.

**Figure 2:**
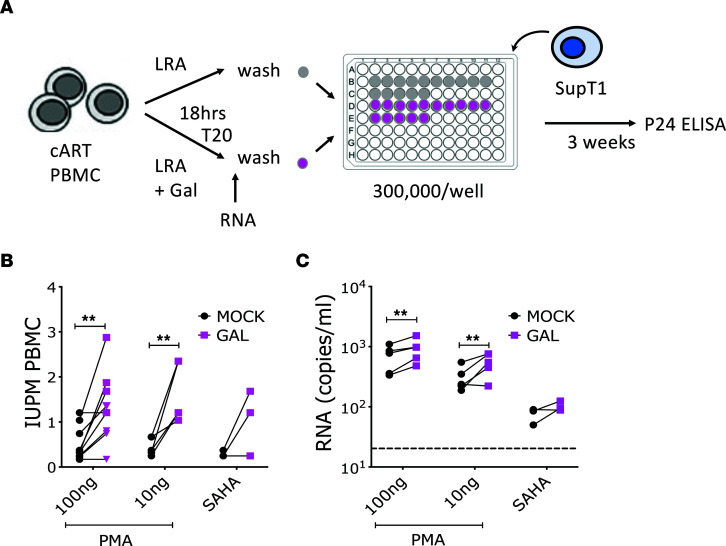
Blocking TGF-β1 signaling increases latency reversal agent–induced HIV-1 reactivation ex vivo. Deidentified PBMCs from aviremic, ART-treated PLWH collected at NIH and Northwestern University were used for viral outgrowth assays to estimate the frequency of HIV-infected cells able to produce replication-competent virus within the unfractionated PBMCs. (**A**) Schematic of the qVOA assay where activation with a latency reversal agent (LRA; namely PMA or vorinostat) was followed by collection of supernatant for viral RNA (vRNA) quantification (*n* = 5) and coculture with SupT1 cells (*n* = 9) in replicate wells (>14 wells). (**B**) The IUPM calculated based on the frequency of p24^+^ wells in each condition are shown (pink squares represent PBMCs from NIH; triangles represent PBMCs from Northwestern, RADAR). (**C**) vRNA-gag copies/mL of culturing media in presence versus absence of galunisertib (1 μM). Conditions were compared by a 2-way ANOVA followed by the Holm-Šídák multiple comparisons test. Significant *P* values of α < 0.05 (*) and α < 0.01 (**) are indicated. All other comparisons were nonsignificant.

Considering that TGF-β is present in FBS used to supplement PBMC and T cell cultures, and that PBMCs from PLWH release TGF-β in culture supernatants (56–58), we hypothesized that blocking TGF-β while reversing HIV-1 latency ex vivo from PBMCs of ART-treated, aviremic individuals may lead to higher frequency of HIV-1 reactivation than in untreated cultures. To test this hypothesis, we used a modified version of the classical quantitative viral outgrowth assay (qVOA) (59). We used whole PBMCs to assess potential latency reactivation from any HIV-1–infected circulating cell and not only from resting CD4^+^ T cells (Figure 2A). PBMCs from 2 different sets of aviremic, ART-treated donors (Table 1) were activated with PMA (at 100 ng/mL or 10 ng/mL) and with vorinostat (SAHA, at 1 μM) in the presence of T20 and galunisertib (1 μM) or mock solution. After overnight incubation supernatants were collected and assayed for both vRNA in a subset of donors (*n* = 5) and viral outgrowth (*n* = 9). After washing out the PMA, cells were plated at 3 × 10^5^/well in 96-well plates with or without galunisertib in replicates (>14 replicate wells), and SupT1 cells were added to amplify the infection. Infectious units per million cells (IUPM) were calculated based on the number of infected wells as determined by p24 ELISA (Figure 2A). A significantly higher frequency of latency reactivation events occurred in the presence of galunisertib in PBMCs from most donors (Figure 2B). This occurred with both high and low PMA concentrations and when reactivation was performed with vorinostat. No other LRAs were tested. Moreover, higher levels of vRNA were detected after PMA stimulation in galunisertib-treated PBMCs than in mock treated (Figure 2C).

To control that addition of galunisertib would not directly impact HIV-1 replication in SupT1 cells or PBMCs, increasing the chances of detecting infection, we performed ex vivo infection of SupT1 cells and PBMCs from healthy donors in the presence of different concentrations of galunisertib (Supplemental Figure 5). Galunisertib had no impact on the HIV-1 growth curves in these in vitro infections.

### Blocking TGF-β1 leads to SIV reactivation in vivo.

**Figure 3:**
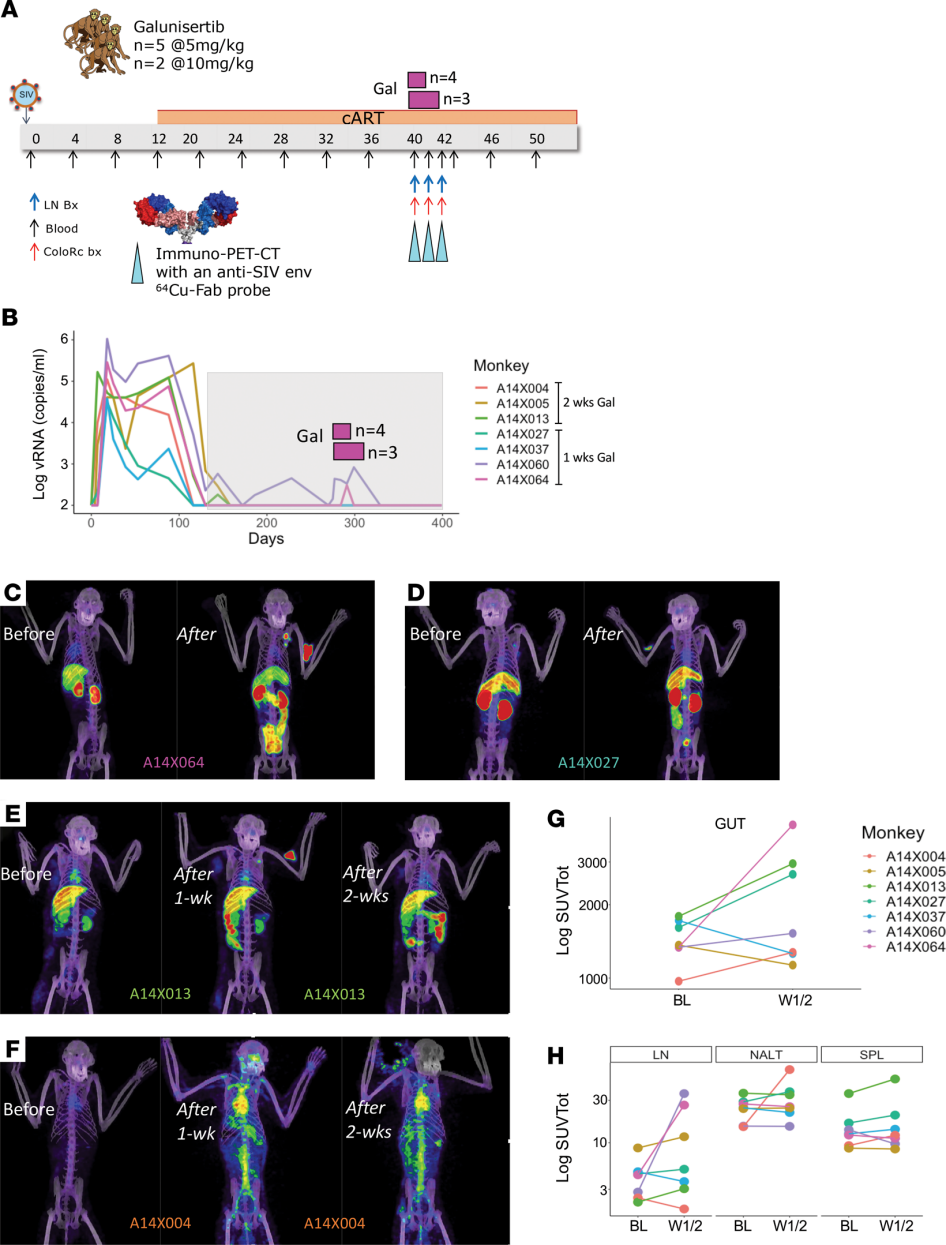
Blocking TGF-β1 leads to HIV-1 reactivation in vivo. (**A**) Schematic representation of the macaque studies. SIVmac251- and SIVmac239-infected macaques (*n* = 7) were treated with antiretroviral therapies starting week 12 postinfection. After 26–27 weeks of ART, animals were treated twice daily with 5 or 10 mg/kg of galunisertib orally. Colorectal biopsies, fine needle aspirates (FNAs), and PET/CT scans with the anti–SIV-env probe ^64^Cu-7D3 were performed before and at the end of the 1 or 2 weeks of treatment. The animals that were treated for 2 weeks underwent a second scan at the end of the first week of treatment. (**B**) pVLs measured at NIRC (lower limit of quantitation = 100 copies/mL). Galunisertib was administered every day (twice daily) for 1 or 2 weeks. Timing of galunisertib administration is represented by pink rectangles above the pVL. (**C**–**F**) Representative images from the PET/CT scans of 4 out of 5 animals with increased PET signal following galunisertib treatment. (**G** and **H**) SUVtot for different anatomical areas (regions of interest) are shown before and after galunisertib treatment (Gut, small and large intestine; LN, axillary LNs; NALT, nasal associated lymphoid tissues; SPL, spleen). Data from baseline (BL) and postgalunisertib weeks 1 and 2 (W1/2) were compared using Wilcoxon matched pairs nonparametric test, and the differences were nonsignificant with α > 0.05.

To test whether galunisertib might facilitate spontaneous episodes of HIV latency reversal, especially in tissues rich in TGF-β such as the gut, we administered galunisertib at 5 mg/kg or 10 mg/kg via oral gavage to 7 SIV-infected, ART-treated macaques (Figure 3A and Table 2). Macaques were infected intrarectally (2,000 tissue culture ID_50_; TCID_50_), or IV (300 TCID_50_) of SIVmac251 or SIVmac239 (Table 2). All macaques but 1 were ARV suppressed (pVL < 100 copies/mL) for at least 100 days before galunisertib treatment. Galunisertib was used at 5–10 mg/kg, which is equivalent to the dose administered in clinical trials for cancer treatment (43, 60) and predicted to be within the therapeutic window in humans by extensive PK/pharmacodynamics (PD) studies (42, 61). PK in rhesus macaques was evaluated in a separate group of naive animals (Table 2) and found to be substantially lower than in humans (Supplemental Figure 6A and ref. 61). However, examination of phosphorylated SMAD2/3 (p-SMAD2/3) over total SMAD in PBMCs suggested that galunisertib was active in the macaques at these concentrations (~25% reduction in p-SMAD2/3, Supplemental Figure 6B).

Galunisertib was administered orally twice per day for 1 week for 4 macaques and 2 weeks for 3 macaques. An increase in pVL over 100 copies/mL was detected in 2 of the 7 macaques (Figure 3B and Table 2). Additional small changes in pVL occurred below 100 copies/mL in the other macaques when pVL was analyzed by ultrasensitive (lower limit of detection 5 copies/mL) assay at Leidos. However, most of the macaques had detectable pVL at baseline when using the ultrasensitive assay, and extensive baseline analysis was not performed to determine whether these changes represented a true deviation from baseline (Supplemental Figure 7).

**Figure 4:**
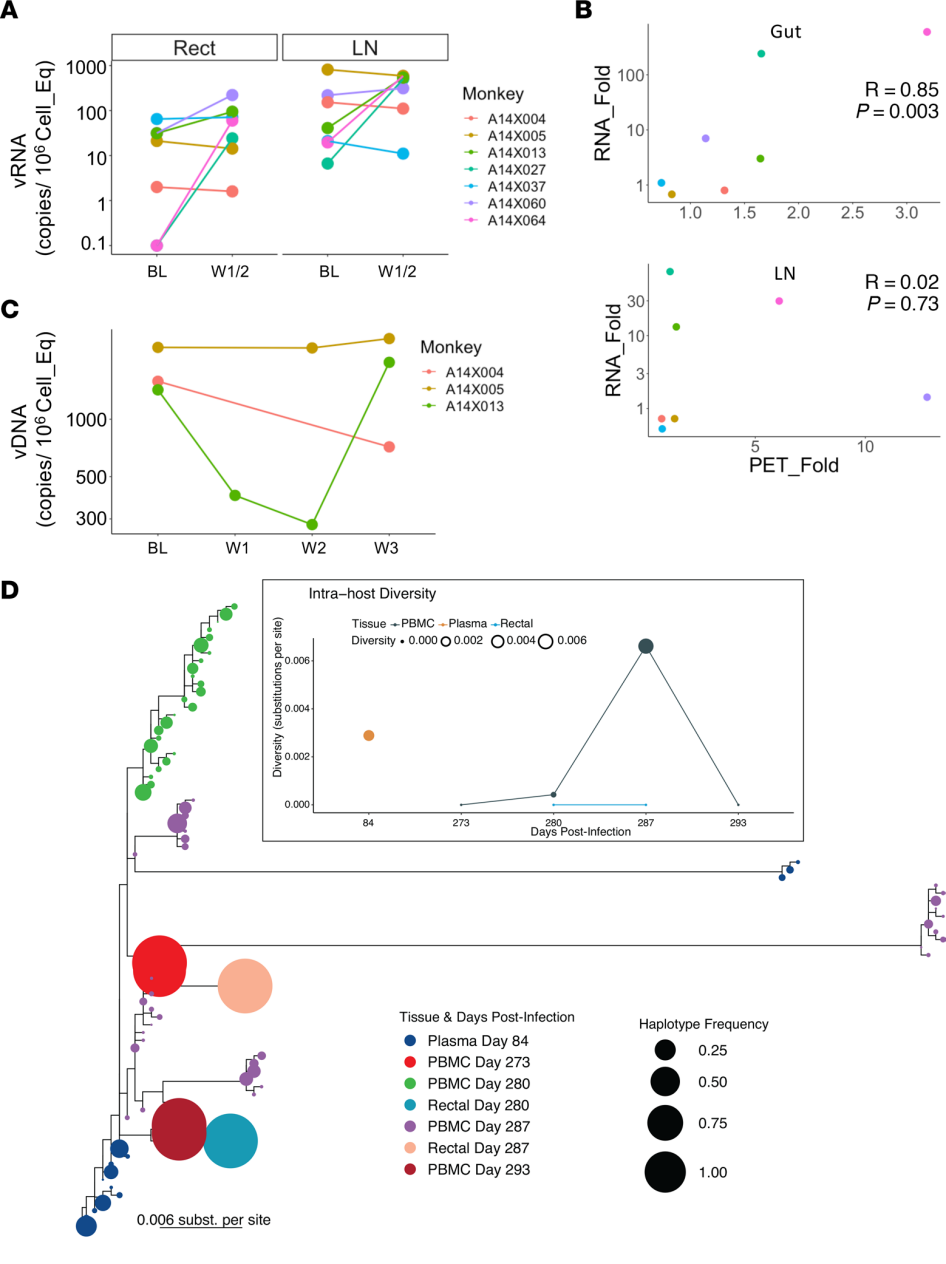
Increased PET signal corresponds to increased vRNA. (**A**) Copies of cell-associated unspliced vRNA normalized on 10^6^ cells diploid genome equivalent are shown for colorectal biopsies and LNAs before and after galunisertib treatment. Data from BL and postgalunisertib W1/2 were compared using Wilcoxon matched pairs nonparametric test, and the differences were nonsignificant with α > 0.05. (**B**) The correlation between the fold increase in SUVtot for the gut (above) and LN (below) and the fold increase in vRNA copies in rectal biopsies and FNAs (only 1 of the axillary LNs was sampled) are shown. Pearson’s correlation coefficient and *P* values are indicated in each graph. (**C**) Copies of vDNA per 10^6^ cells equivalent are shown for the PBMCs of the 3 animals that were treated with galunisertib for 2 weeks. W3 time point represents a sample collected 7 days after the last galunisertib administration. No statistical test was performed. (**D**) Intrahost viral quasispecies evolution before and after galunisertib treatment in A14X013 is shown. Inferred maximum likelihood (ML) tree with quasispecies within the same host in PBMCs, rectal tissues, and blood samples using deep sequencing of the gag gene. Circles at the tips represent each haplotype and are colored by time postinfection and sample type (D84 is plasma right before ART initiation; D273 is PBMC on the day of first galunisertib treatment, collected before treatment; D280-287-290 represent 7, 14, and 20 days after galunisertib initiation). Circle size indicates the frequency of the haplotype in the quasispecies. Insert represents the spatiotemporal evolution of the viral population diversity at each compartment and time point. Both *y* axis and circle size indicate diversity of quasispecies, and data from the same sample type are connected by lines. Intrahost viral diversity was calculated as weighted average of pairwise distances between every haplotype weighted by their frequency in the population in substitutions per site.

ImmunoPET/CT images with the validated ^64^Cu-DOTA-F(ab′)_2_ p7D3 anti–SIV-env probe (62, 63), colorectal biopsies (bx), lymph node (LN) FNAs, and blood samples were collected at baseline (day –1 of treatment) and on day 7 or 14 of treatment. Notably, a clear, extensive viral reactivation occurred in the gut of 3 of the 7 macaques as documented by the notable increase in PET signal (A14X064, A14X027, and A14X013; Figure 3, C–E; and Supplemental Videos 1, 4, and 7). A smaller increase in the gut was also present in A14X004 and A14X060 (Figure 3G, Supplemental Video 5, and Supplemental Figure 1). An increase in PET signal also occurred in the LNs of A14X064, A14X013, and A14X060 (Figure 3, C and E; and Supplemental Videos 3, 4, and 7) and nasal associated lymphoid tissues, the spinal cord, and the area surrounding the heart (A14X004, Figure 3F and Supplemental Video 5). These increases were captured by the change in total standard uptake values (SUVtots) in the different anatomical areas as summarized in Figure 3, G and H. No reactivation was detected in macaques A14X037 and A14X005 (Supplemental Videos 2 and 6). The ^64^Cu-DOTA-F(ab′)_2_ p7D3 was previously validated in SIV-uninfected macaques (62). However, a probe generated using a rhesus IgG1 Fab against an irrelevant antigen ^64^Cu-DOTA-F(ab′)_2_ pIgG1 in an SIV-infected macaque was used as further control (Supplemental Figure 8 and Supplemental Video 2). Moreover, a resection of the PET-positive area in the small intestine of an SIV-infected macaque was used as control of the association between the PET signal and the level of SIV replication in a specific tissue (Supplemental Figure 9),

Viral reactivation in the macaques was supported by large (1 to 3 log) increases in cell-associated vRNA (CA-vRNA) observed when colorectal bx and LN FNAs were assayed for RNA (Figure 4A). Notably, the increase in rectal biopsy CA-vRNA correlated well (*R* = 0.85; Figure 4B) with the increase in SUVtot in the gut region. This occurred even though bx were collected from the colorectal area while the SUVtots for the gut represent the signal in the entire intestine. In contrast, no correlation was present between vRNA in the FNAs that sampled a single LN (Table 2) and the SUVtot signal in both axillary LNs (Figure 4C).

In some macaques vRNA increase was paralleled by a small increase in CA-vDNA (Supplemental Figure 10A; e.g., in A14X060 for colorectal bx and A14X027 and A14X064 for LNs). In other macaques CA-vRNA increase was not accompanied by an increase in CA-vDNA, and CA-vDNA varied substantially between baseline and after galunisertib treatment. However, the CA-vRNA/vDNA ratio increased substantially in at least 2 macaques (A14X027 and A14X064, Supplemental Figure 10B).

We examined the levels of CA-vDNA in the PBMCs of the 3 macaques that were treated for 2 weeks (samples were not available for the animals treated for 1 week). Interestingly, we noted a drop in the CA-vDNA levels in the PBMCs of A14X013. Since this macaque had a strong viral reactivation signal via PET (Figure 3E), we considered this drop interesting and worth further evaluation by viral sequencing. The increase in CA-vRNA in the tissues of this macaque was balanced by an increase in CA-vDNA, suggesting that galunisertib may have induced recirculation of infected cells from blood to tissues and vice versa. This was supported by the sequencing data that demonstrated a substantial increase in the diversification of the intrahost viral quasispecies obtained from A14X013 PBMCs during the 2 weeks of galunisertib treatment (Figure 4D). The inferred phylogeny of the intrahost quasispecies at the different time points and anatomical locations suggests the expansion of multiple viral lineages at week 1 and 2 after galunisertib initiation in PBMCs (Figure 4D). Viral diversity was low at the time of galunisertib initiation in PBMCs. This was followed by high levels of diversification in blood while observing compartmentalization between PBMCs and rectal tissues that additionally displayed low-diversity populations. At week 3 after galunisertib treatment initiation (1 week after the end of the treatment), viral diversity in blood dropped, and the new dominant viral populations in blood were closely related to the later viral populations in rectal tissues detected a week earlier. Overall, this suggests a substantial viral expansion during galunisertib treatment of multiple infection foci and migration of viral populations between tissue and blood in A14X013.

Interestingly, we observed an increase in the levels of TGF-β in blood after galunisertib treatment (Supplemental Figure 11A). However, reactivation was not linked to baseline concentrations of circulating total TGF-β (Supplemental Figure 11B).

### Blocking TGF-β1 enhances SIV-specific responses.

**Figure 5:**
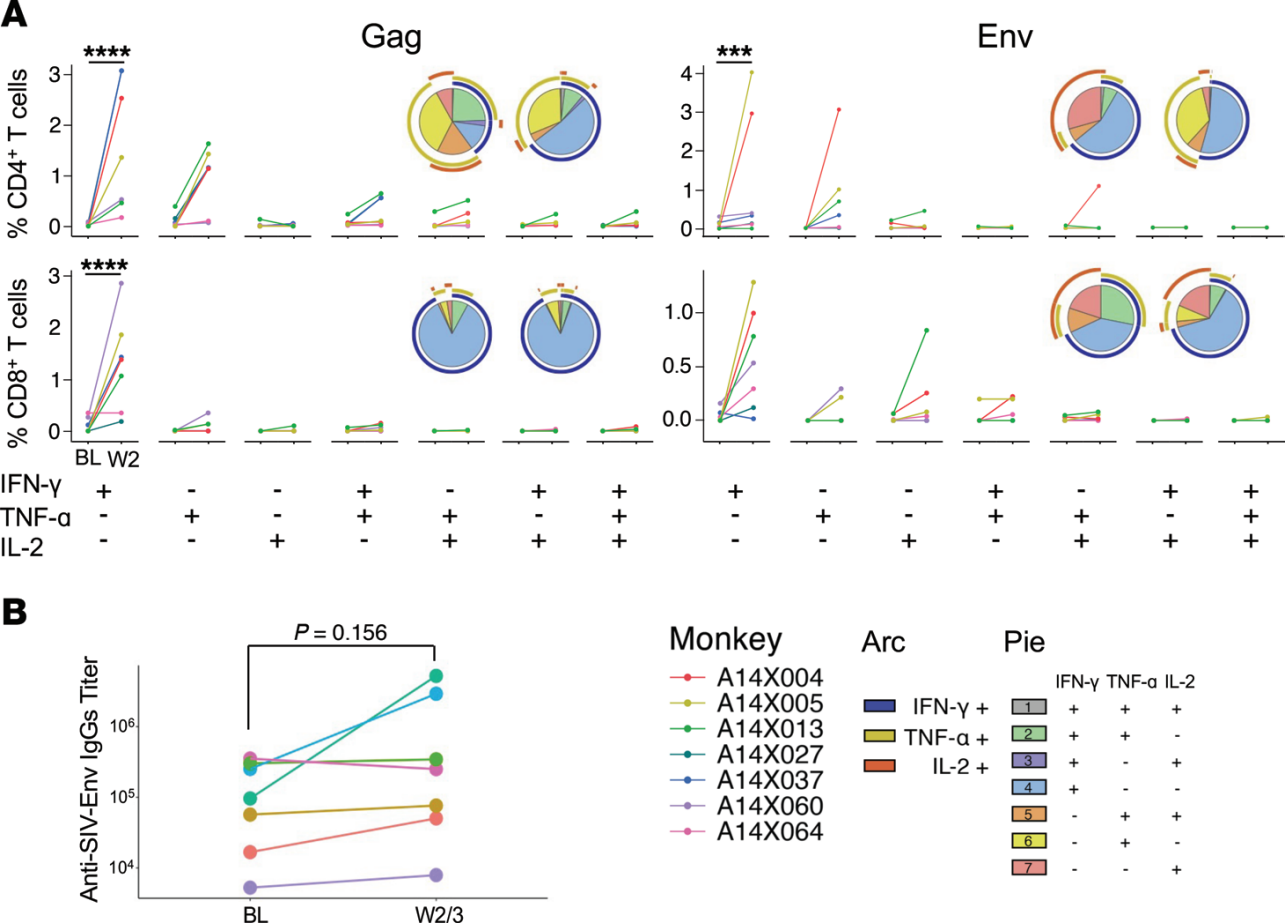
Blocking TGF-β1 stimulates SIV-specific responses. (**A**) The frequency of CD4^+^ T cells and CD8^+^ T cells that express the cytokines indicated below the graphs in response to stimulation with a pool of 15-mer overlapping peptides from SIVmac239 gag and env are shown for before and after galunisertib treatment. The frequency of each population was calculated after subtraction of a DMSO unstimulated control performed in parallel with each sample. A mixed-effect model with the Holm-Šídák multiple comparisons test, with a single pooled variant, was used to test for significant differences. Significant *P* values of α < 0.001 (***) and α < 0.0001 (****) are indicated. Only IFN-γ was significant with this test. Nonsignificant differences in all other cytokine combinations are not indicated. (**B**) Anti–SIV-env titers in plasma before and after galunisertib treatment are shown for each animal. Wilcoxon matched pairs nonparametric *t* test was used to compare before and after galunisertib.

Since TGF-β is primarily an immunosuppressive factor, blocking TGF-β1 signaling was expected to impact phenotype and function of immune cells independent of SIV infection. Indeed, a preliminary mRNA-Seq analysis of CD4^+^ T cells from before and after (6 hours) galunisertib treatment in 3 naive macaques suggested several changes in pathways regulating immune functions, such as leukocyte activation and adaptive immune system (downregulated) and regulation of DNA binding, MAPK, and FAK pathways and TCR signaling (upregulated) (Supplemental Figure 12). Hence, we investigated the impact of TGF-β1 blockade with galunisertib on SIV-specific T cell and B cell responses. T cell responses were probed via classical PBMC stimulation with gag and env peptide antigen followed by detection of intracellular IFN-γ, TNF-α, and IL-2 by flow cytometry. We found a significant increase in the frequency of IFN-γ–producing CD4^+^ and CD8^+^ T cells following both gag and env peptide stimulation (Figure 5A). However, we observed no increase in the frequency of cells producing multiple cytokines (pluripotent cells). Subset analysis using a CD95 memory marker was not performed.

Finally, in 3 of the 7 macaques, there was a notable (0.5 to 1 log) increase in the titer of SIV-env–specific antibodies following galunisertib treatment (Figure 5B). However, this increase did not occur in macaques with large viral reactivation as measured by SUVtot increase. Rather antibody titers increased in animals with no change (A14X037) or relatively lower increase (A14X027 and A14X004) in gut SUVtot (Figure 3G).

## Discussion

HIV-1 persists in different cell types during ART, and it is now clear that the mechanisms of viral persistence are heterogeneous. HIV-1 transcriptional activity varies in different cell types and is dependent, at least in part, on tissue location and environmental cues. HIV-1 latency in T cells is maintained through diverse mechanisms that include blocks in transcriptional elongation, completion, and splicing (2). The differential role of these blocks on HIV-1 expression in T cells depends on the tissue and microenvironment where infected cells reside, and the same factors may also influence the ability of HIV-1 to emerge from latency (64, 65). Similar heterogeneous, tissue/environment-dependent mechanisms may be at play in regulating HIV-1 replication in other HIV-1 cell reservoir types, such as myeloid cells. Finally, the long-term persistence of these infected cells may be dependent on homeostatic (66, 67) or antigen-driven proliferation (3, 68), with a potential contribution of promoter insertion mutagenesis (69, 70). The different cellular processes that regulate the dynamics of the HIV-1 reservoir remain to be fully clarified. However, a common characteristic shared by HIV-1 target cells of both T and myeloid cell lineages that constitute the HIV-1 “invisible” reservoir is their “resting” phenotype. TGF-β represents a common factor released at high level in PLWH. It limits the ability of HIV-1–infected cells to respond to activation stimuli and raises the threshold for TCR-driven proliferation (71) while not fully impairing homeostatic proliferation (72). The capacity of TGF-β to broadly inhibit cell activation in several different potential HIV-1 cellular reservoirs, while not fully blocking the ability of infected cells to proliferate, led us to test the hypothesis that TGF-β might be a major factor that contributes to the low inducibility of intact proviral sequences in a wide range of cell types (13). We reasoned that inhibiting TGF-β signaling might decrease the threshold for transcription and translation of HIV-1 proteins in cells activated by different stimuli both ex vivo (in the presence of exogenous TGF-β1, including the TGF-β1 present in FBS; ref. 21) and in vivo. In tissues, especially mucosal tissues such as the gut, TGF-β is a major player and regulator of tissue homeostasis (73). Moreover, the gut is a site of constant antigenic exposure. This is especially true in HIV-infected individuals, in which compromised gut barrier function is a source of inflammatory stimuli that likely drives stochastic HIV-1 reactivation. Indeed, in vitro, using the classical cell line models ACH-2 and U1, we demonstrated that addition of exogenous TGF-β decreased the ability of PMA, a powerful PKC activator, to induce HIV-1 reactivation, and as a consequence, fewer cells expressed HIV antigens in the presence of TGF-β. This effect was blunted when TGF-β was added in the presence of galunisertib. In some cases, we detected enhanced HIV-1 reactivation in the presence of galunisertib alone, which likely reflected galunisertib-mediated inhibition of FBS-derived TGF-β.

We also demonstrated that TGF-β acts both directly and indirectly on HIV-1–infected cells. Indeed, TGF-β–treated moDCs exhibited a reduced ability to reactivate HIV-1 from latently infected T cells. Historically, PBMCs and CD4^+^ T cells have been cultured in the presence of FBS. Both FBS-derived TGF-β and TGF-β released by HIV-infected PBMCs likely exert an inhibitory effect on HIV reactivation ex vivo in qVOA-type assays. This explains the activity of galunisertib in increasing the frequency of latency reactivation in our qVOA. Importantly, our qVOA was a highly simplified and modified version of the classic qVOA (59). A major limitation of this work is that we have only tested PMA and vorinostat as LRAs. We did not determine whether inhibiting TGF-β signaling may also increase HIV reactivation following treatment with BRD4 inhibitors, HMT inhibitors, or TLR inhibitors. TGF-β1 is known to inhibit TLR signaling, so it is likely that galunisertib will enhance the LRA activity of TLR agonists. Its impact on other LRA classes is less clear and will need to be addressed in future studies.

The most intriguing result that we obtained involved the ability of galunisertib to drive SIV reactivation in vivo in aviremic, SIV-infected macaques. While we detected a clear SIV-env signal in tissues by PET/CT following galunisertib treatment in 5 out of 7 animals, we detected an increase in VL in only 2 out of the 7 animals. A14X060, whose viremia had not been continuously suppressed in blood before galunisertib treatment, had a very high (>2 log) increase in pVL with a relatively low increase in gut SUVtot. However, this macaque had a substantial increase in PET signal in the LNs, which may explain the increase in pVL. In contrast, in the other 4 macaques that showed reactivation in tissue, there was minimal or no increase in vRNA in blood. Considering that we did not sample blood every day, rather every 3–4 days, it is possible that we missed a transient increase in pVL. However, it is also possible that viral expression/reactivation was localized in tissues and not enough virus reached the blood to be detected. Importantly, the observed increase in SUVtot in the gut was reflected in the increase in vRNA in colorectal bx. This occurred despite the highly focalized sampling of bx compared with measuring the overall PET signal in the entire organ, warranting further study and confirmation. However, it is important that the data collected with the 2 techniques agreed with each other. In contrast, the lack of correlation between increase in PET signal in LNs and the increase in vRNA in FNAs may be due to the FNA technique involving sampling a single site, not necessarily axillary, while the PET signal that we could isolate with the MIM software encompassed only the axillary LNs. A further confirmation of the value of the increase in PET signal in specific tissue area is given by the analysis of a gut tissue resection that captured an area of PET signal in an SIVmac239-infected macaque (Supplemental Figure 9). The SUVtot in each tissue area correlated with the amount of vRNA detected in that part of the tissue. The sensitivity of the ^64^Cu-DOTA-F(ab′)_2_ p7D3 probe has not been evaluated in vivo because of its dependency not only on the number of SIV-gp120–expressing cells,but also on the amount of gp120 expression and cell density within a specific tissue volume. However, Supplemental Figure 9 demonstrates that immunoPET/CT can capture small differences in viral replication at least as well as reverse transcription quantitative PCR (RT-qPCR) would.

Some insights into the dynamics of viral rebound on ART during galunisertib treatment come from the sequencing done on A14X013. This animal had a large increase in gut SUVtot detected by immunoPET, but no increase in pVL was detected with our sampling schedule. Yet we found evidence of increased viral diversity during galunisertib treatment particularly in blood that concluded with a new dominant viral population closely related to those detected in rectal tissue. Although this analysis was done only in 1 macaque, it suggests that galunisertib may induce viral expansion of multiple infection foci and migration of viral populations between tissue and blood.

Finally, we observed an increase in SIV-specific immune responses that appeared to be driven by the galunisertib treatment. Since these increases were also detected in the animals that exhibited no detectable rebound (A14X005 and A14X037), they may not have been directly related to increases in viral antigen. This is intriguing and suggests potential direct effect of inhibiting TGFBR1 signaling on SIV-specific CD4^+^ and CD8^+^ cells. An increase in T cell function was also detected during the preclinical and clinical development of galunisertib (41, 42). Interestingly, the 2 animals in which reactivation could not be detected had the highest increase in SIV-specific antibody responses. This suggests that the increased SIV responses may have masked SIV reactivation in these animals.

Whether this increased immune response could translate into increased purging of the viral reservoir was not the focus of this initial proof-of-concept study and remains to be determined. In any case, the combination of viral reactivation and enhanced cellular and humoral responses in this pilot study is highly promising and suggests that galunisertib may have an impact on the viral reservoir alone or in combination with other strategies to enhance immune functions killing virus-expressing cells.

The studies presented herein have several limitations. They include the use of cell lines as models for HIV latency, which may not fully recapitulate the mechanisms of latency in vivo. Moreover, as mentioned above, only PMA and SAHA were tested in combination with galunisertib in a limited number of PBMC samples from aviremic PLWH. Additional studies will be needed to dissect potential differences in the activity of galunisertib on different mechanisms of HIV-1 reactivation. Regarding the in vivo studies, the limited number of animals and lack of prolonged follow-up or ART interruption as well as relatively short treatment course are limitations that can be addressed in future studies now that we have determined that galunisertib is able to mediate virus reactivation. Our dosing was based on preclinical studies in other animals and on the therapeutic index used for clinical studies in humans. However, the concentration of galunisertib in the blood of macaques is at least 10-fold lower than the average concentration detected in human plasma after similar dosing. Future work will require more detailed PK/PD studies in macaques. Another drawback of our study is that we are missing analysis of cell phenotypes in PBMCs and tissues before and after galunisertib administration in vivo. This was not done because of limited sample availability. In future studies, it will be important to determine whether galunisertib treatment leads to an increase in markers of immune activation or an increase in the expression of inflammatory markers. Finally, virus sequencing was limited to 1 macaque and to gag. This limits our ability to draw any conclusion on the effect of galunisertib on viral dynamics. Near-full-length viral sequencing should be performed in future studies in all animals to better clarify the impact of galunisertib on viral dynamics during ART. It is noteworthy that viral reactivation did not correlate with higher baseline TGF-β concentrations in blood. Hence, the effect of galunisertib on the virus appears to be independent of TGF-β levels in plasma, and the levels in plasma may not directly reflect the levels in tissues. Moreover, we monitored total TGF-β, and the levels of active TGF-β may be different. Future studies should include monitoring of TGF-β in tissue at baseline to understand whether galunisertib may work better in the context of high baseline tissue TGF-β levels.

In conclusion, we have demonstrated that TGF-β inhibits HIV reactivation from latently infected cells, an activity that may impact ex vivo assays aimed at quantifying the size and dynamics of reservoirs. We further demonstrate that a small-molecule TGFBR1 inhibitor, galunisertib, in advanced stages of clinical development, can promote viral reactivation ex vivo and in vivo. Measurements of pVL did not detect reactivation in vivo in all instances, likely due to insufficient sensitivity. Our work demonstrates that immunoPET/CT imaging can provide important additional information regarding the efficacy of HIV-1 cure strategies. Because this was a first attempt at using an antagonist of TGF-β to reactivate the HIV-1 reservoir in vivo, we expect that optimization of treatment protocols will lead to improved activity. Nonetheless, our results are both surprising and promising and may lead to novel therapeutic interventions to reduce or eliminate the HIV-1 reservoir and bring the field closer to a cure.

## Methods

### Study design.

A total of 15 adult male and female Indian rhesus macaques (*Macaca mulatta*; Mamu A*01, B*08, and B*17 negative) were used for the in vivo studies described (Table 2). Two additional rhesus macaques, A8L057 and 08M171, were infected with SIVmac239M2 (provided by Brandon Keele, Frederick National Lab, Leidos Biomedical Research, Frederick, Maryland, USA) and used as controls for, respectively, Supplemental Figures 8 and 9 at, respectively, weeks 56 and 32 postinfection. All rhesus macaques used in these studies were selected from the colonies bred and raised at the NIRC, University of Louisiana at Lafayette. Treatment of all macaques were in accordance with NIRC standards and any abnormal observations and/or signs of illness or distress were reported per NIRC standard operating procedures. All efforts were made to minimize suffering and stress. Appropriate procedures were performed to ensure that potential distress, pain, and discomfort were limited to that unavoidable in the conduct of the research plan. Ketamine (10 mg/kg) and/or telazol (4 mg/kg) were used for sample administration, collection of samples, and conducting image collections. Analgesics were used when deemed appropriate by veterinary medical staff. Monkeys were fed monkey chow supplemented with fresh fruit or vegetables and water ad libitum.

Rhesus macaques (*n* = 7) were infected with SIVmac251 or SIVmac239 rectally or IV (Table 2), and ART (tenofovir, emtricitabine, and dolutegravir) was initiated between week 12 and 17 postinfection. Galunisertib treatment was initiated after 26 or 27 weeks on ART for 7 or 14 days. Galunisertib powder (MedChemExpress) was dissolved in water and given orally in a treat twice daily at 5 mg/kg or 10 mg/kg (Table 2). Blood VL was monitored biweekly before and during ART and every 3–4 days during galunisertib treatment. Colorectal bx and LN FNAs were collected before and after galunisertib treatment.

PET/CT imaging with a ^64^Cu-DOTA-F(ab′)_2_ p7D3 anti–SIV-env probe was performed before galunisertib treatment, after 7 days in all macaques, and after 14 days in the macaques that were treated for 14 days. Briefly, a solution of ^64^Cu was incubated with DOTA-F(ab′)_2_ p7D3 antibody (produced in-house at NIRC under NIH R240D010947) reconstituted with 50 μL of 1 M NH_4_OAc, pH of 5.5; incubated for 1 hour at 37°C; then purified by ultrafiltration in a desalting spin column (10,000 MW cutoff, Thermo Fisher Scientific); and finally eluted with sterile saline prior to injection. The labeled probe was injected IV via catheters, 24 hours before PET/CT scanning. For taking PET/CT scans, the macaques were sedated with ketamine HCl (10 mg/kg), and/or telazol (4–8 mg/kg), intramuscularly, and supplemented with ketamine (5 mg/kg) as needed to perform the procedure. The macaque’s body was immobilized in dorsal recumbency on the scanner table, and PET/CT scans were acquired using a Philips Gemini TF64 PET/CT scanner. The final CT image was compiled from 250 to 300 slices, depending on macaque size.

PET image analysis was performed using the MIM software. PET/CT fusions were generated and scaled according to calculated SUVs. The SUV scale for the PET scans was selected based on the overall signal intensity of the PET scans (whole body), and the CT scale was selected for optimal visibility of the tissues. All images and maximum image projections were set to the same scale for visual comparisons.

Regions of interest (ROIs) were isolated using a combination of the Region Grow function and manual contouring on a representative scan. These regions were then copied onto subsequent scans of the same animal using a specialized developed workflow. This workflow uses the CT scans to map the selected ROI and locate that corresponding volume in subsequent scans. Manual adjustments were then used to counter any changes in the animals’ orientation between scans. The signals of the whole body as well as within these ROIs were quantified, and max, mean, and SUVtots were calculated for further analyses. Organs of high and unwanted signal (kidneys and liver) were masked using the Region Grow function for selecting the boundaries of the organ signal and the Mask function to reduce the signal within the contour to background levels (for A14X004 only).

### SIVmac251 and SIVmac239 preparation and administration.

Viral stocks were propagated and titrated in rhesus PBMCs and prepared at NIRC for either intrarectal or IV dosing in a biosafety cabinet and diluted in medium. Rhesus macaques were inoculated IV with 300 TCID_50_ of SIVmac239 or intrarectally with 2,000 TCID_50_ of SIVmac251 or SIVmac239. IV injections or intrarectal administration were done using 1 mL of diluted virus while macaques were under sedation.

### In vitro models of HIV latency reactivation.

ACH-2 cells and U1 cells were obtained through the NIH HIV Reagent Program, Division of AIDS, NIAID, NIH (contributed by Thomas Folks). Cells (10^5^/well) were cultured in triplicates in RPMI with 10% FBS and penicillin/streptomycin in the presence of TGF-β1 (10 ng/mL, R&D Systems), TGF-β1, and galunisertib (1 μM MedChemExpress); with galunisertib alone; or with mock treatment. In the HIV latency reactivation conditions, PMA (MilliporeSigma) was added at 100 ng/mL together with galunisertib or TGF-β1. After 18 hours, cells were stained intracellularly for p24 using the KC57 antibody (NIH HIV Reagent Program) as previously described (74).

Blood from deidentified donors was obtained from the New York Blood Bank and used as a source of CD4^+^ T cells for the primary model of HIV latency in vitro. Total CD4^+^ T cells were isolated with the CD4^+^ T cell isolation kit (Miltenyi Biotec) and activated with 5 μg/mL of PHA and 50 U/mL of IL-2 for 3 days. After washing out the PHA, cells were infected with 200 TCID_50_ of HIV ADA (NIH HIV Reagent Program) in 12- or 24-well plates and spinoculated at 1,200*g* for 2 hours at 25°C. After an additional 3 hours of incubation at 37°C, viral inoculum was washed, and cells were plated in the presence of T20 (enfuvirtide acetate salt, 1 μM, NIH HIV Reagent Program). After 48 hours, cells were treated with PMA (100 ng/mL) in the presence or absence of galunisertib (1 μM) overnight and stained for p24 intracellularly as described above.

CD14^+^ cells were isolated from PBMCs using the Miltenyi Biotec CD14^+^ beads. moDCs were obtained from CD14^+^ cells cultured in GM-CSF and IL-4 as previously described (55). At day 4 of moDCs’ differentiation, TGF-β1 (10 ng/mL, R&D Systems) was added to the moDCs to generate the TGF-β–DCs (or both TGF-β1 and galunisertib). At day 6, 2 days after TGF-β1 addition, moDCs were phenotyped (in 3 donors; see Supplemental Table 1 for antibody list) and cocultured with eFluor670-labeled (eBioscience) U1 at 3:1 U1/DC ratio (5 donors). After 18 hours of coculture, cells were stained for p24 intracellularly as described above.

### qVOA.

The standard qVOA assay (59) was extensively modified. PBMCs from aviremic donors were used in the absence of resting CD4^+^ T cells’ isolation. Deidentified PBMCs were obtained either from NIAID protocol 14-I-0039 or 10-I-N048 or from the Northwestern University RADAR cohort.

PBMCs were thawed and cultured in RPMI with 10% FBS at 3 × 10^6^/mL. Cells were activated with PMA (100 ng/mL or 10 ng/mL) or vorinostat (1 μM, NIH HIV Reagent Program) in the presence of T20 (NIH HIV Reagent Program) and IL-2 (100 U/mL) and galunisertib (1 μM) or mock treatment (DMSO). After 18 hours, cells were washed twice in PBS and plated at 3 × 10^5^/well (>14 replicate wells; a number where we expected <1 HIV latently infected cell/well; ref. 75) in a flat-bottom, 96-well plate. 10^5^ SupT1-R5 (M10 clone, NIH HIV Reagent Program) were added in each well, and galunisertib (1 μM) or an equal volume of DMSO was added to the respective wells in each condition. A total of 50 μL of supernatant was collected after 7, 14, and 21 days of coculture and monitored for p24 presence by p24 ELISA (PerkinElmer). IUPM were calculated based on the number of positive wells using the IUPM calculator provided (76). Supernatant from stimulated PBMCs before coculture with SupT1 cells was tested by *gag*–RT-qPCR (77).

For control experiments of the impact of galunisertib on HIV infection of SupT1 cells and PBMCs, SupT1 cells and PHA-activated PBMCs were cultured, respectively, at 1 and 2 million/mL in R10 (in the presence of 20 U/mL of IL-2 for PBMCs) and infected with 50 TCID_50_/10^6^ cells of HIV-Bal (0.01 MOI). HIV replication was monitored by p24 ELISA.

### Plasma and tissue SIV VLs.

Blood was collected in EDTA tubes and plasma was separated by density gradient centrifugation and used for the determination of plasma VLs by SIVgag RT-qPCR at NIRC or at Leidos (Quantitative Molecular Diagnostics Core, AIDS and Cancer Virus Program, Frederick National Laboratory). Tissue VLs from snap-frozen colorectal bx and LN FNAs were performed at Leidos (A14X004, A14X005, and A14X013) as described (78) or, for the remaining macaques and from PBMCs of all macaques, they were performed as described (77).

### Galunisertib PK and PD in macaque blood.

We used 8 naive macaques (Table 2) to collect plasma and PBMCs for PK/PD studies. Of them, 4 macaques received galunisertib (2.5 mg/kg, *n* = 2, or 5 mg/kg, *n* = 2) once, and blood was collected at 1 hour, 3 hours and 6 hours, and 24 hours and 48 hours. The other 4 macaques received galunisertib (2.5 mg/kg, *n* = 2, or 5 mg/kg, *n* = 2) twice daily for 9 days, and samples were collected 1 hour, 3 hours, and 8 hours after administration on day 1 and on day 9 right before and 1 hour and 3 and 6 hours after the last administration. Plasma concentrations of galunisertib were measured by IMSERC utilizing liquid chromatography–mass spectrometry (LCMS, Shimadzu UHPLC system and SCIEX Qtrap 6500+ triple-quadrupole mass spectrometer). All standard, quality control (QC), and experimental samples were prepared and ran in duplicate using 20 μL of plasma (spiked with galunisertib if a standard or QC), adding 15 μL of 8% phosphoric acid solution and 200 μL of acetonitrile (to crash plasma proteins) spiked with tramadol as an internal standard. The standard curve was prepared as a 10-point curve from 1.0 to 1,000 ng/mL with 3 QCs. For the LCMS method, a 1 μL injection was performed of the resulting supernatant separated on a Phenomenex Kinetex Biphenyl 2.6 μm 100A LC column (50 × 2.1 mm). The separation gradient was performed using 0.1% formic acid in water (A) and 0.1% formic acid in methanol (B) mobile phases following a gradient at 0.6 mL/min flow rate of 0.5 minutes 10% B, 4.0 minutes 85% B and hold for 1.0 minutes, and returning to 10% B at 5.1 minutes holding for 1.4 minutes. All samples and standards were processed in duplicate with the average AUC being used for quantitation reporting. After internal standard correction, all samples and standards outside of a 20% coefficient of variation between preparation replicates were rerun in duplicate to meet this requirement. The resulting limit of quantitation was 1.0 ng/mL.

For PD studies, the levels of p-SMAD2/3 were measured using the PathScan PhospoSMAD2/3 ELISA kit (Cell Signaling Technology) over the levels of total SMAD2/3 in cell lysates with Total SMAD ELISA Kit (Cell Signaling Technology) normalized on protein content.

### Sequencing of SIV genome in PBMCs, rectal bx, and plasma from A14X013.

DNA was extracted from PBMCs and rectal bx using the DNeasy Blood & Tissue Kit (QIAGEN), while RNA was extracted from plasma using the QIAamp Viral RNA Mini Kit (QIAGEN). Extracted RNA was immediately reverse-transcribed using SuperScript VILO cDNA Synthesis Kit (Invitrogen). Q5 Hot Start High-Fidelity 2X Master Mix (New England Biolabs) was used to amplify the *gag* gene with primers Outer-Gag-F (5′ GAAGCAGGAAAATCCCTAGCAG 3′) and 8R (5′ CCAACTGACCATCCTTTTCCATCTTT 3′) followed by a second amplification round using primers 1F (5′ TCCTGAGTACGGCTGAGTGAAG 3′) and 7R (5′ TCCTATTCCTCCTACTATTTTTGGGGT 3′) (79). PCR cycling conditions were as follows: 98°C for 30 seconds, 35 cycles of 15 seconds at 95°C, 30 seconds at 65°C, 2 minutes at 72°C, and a final extension of 72°C for 2 minutes. Sequencing library preparation of the obtained amplicons was performed using the SeqWell plexWell 384 kit per manufacturer’s instructions. Pooled libraries were sequenced on the Illumina MiSeq using the V2 500 cycle kit. We subsequently performed reference-based mapping assembly of the sequencing reads using the HAPHPIPE protocol and SIVmac239 reference genome (M33262.1). With this protocol, reads were trimmed to remove adapters and low-quality sequences using Trimmomatic v0.39 and error-corrected using SPAdes v3.13.1. PBMC samples were sequenced twice to confirm the results obtained. Sequences were deposited in GenBank: OP390088-OP390161.

### Phylogenetic and diversity analysis.

With the assembled reads we performed probabilistic inference of intrahost viral quasispecies for each sample obtained from A14X013 using QuasiRecomb. The sequences of the inferred viral haplotypes from each quasispecies were aligned using MAFFT v7.453 software, and an ML phylogeny was reconstructed with IQ-Tree v2.0.5 to track the spatiotemporal evolution of the intrahost viral populations. The final tree representation was performed with the R package ggtree v3.2.1. Intrahost diversity for each sample analyzed was calculated using DistanceCalculator in Biopython and pairwise distances weighted by the estimated frequency of the haplotype with an in-house python script.

### Anti-SIV T cell responses.

Frozen PBMCs collected right before the first galunisertib administration and right after were thawed in AIM V medium (Thermo Fisher Scientific) plated on a plate precoated with 2.5 μg/mL goat anti-mouse IgGs (AQ127 MilliporeSigma) and cross-linked with 10 μg/mL anti-CD28 and anti-CD49d antibodies (MilliporeSigma clones CD28.2 and HP2/1). Cells were stimulated either with SIVMAC239 GAG peptide pool (2 μg/mL) or with SIVMAC239 ENV peptide pool (2 μg/mL HIV Reagent Program, Division of AIDS, NIAID, NIH) or the same volume of DMSO as baseline control. Pooled cells stimulated with 1 μg/mL PMA/ionomycin were used a positive activation control. One hour later 10 μg/mL Brefeldin A and monensin (GolgiStop, BD Biosciences) were added to each well. After 5 hours, cells were transferred to a FACS plate and stained with the LIVE/DEAD Aqua viability dye (Thermo Fisher Scientific) and antibodies against cell surface and cytokines (Supplemental Table 1).

### Anti-env antibodies.

We coated 96-well plates with the SIV gp130 recombinant protein (HIV Reagent Program, Division of AIDS, NIAID, NIH) at 1 μg/mL in coating carbonate buffer 2 hours at room temperature. After washing the plates 4 times with washing buffer, we incubated the plates in blocking buffer (2% BSA in PBS) overnight. Plates were washed and dilutions of test sera or control sera were added to the plate starting at 1:50. Sera were incubated at 37°C for 30 minutes and plates washed and detected with anti-rhesus IgG HRP (Nonhuman Primate Reagent Resource) 30 minutes at 37°C. After washing and substrate incubation at room temperature for 30 minutes plates were read at 450/650 nm.

### Statistics.

Data from different conditions of ACH-2 and U1 experiments were compared using the Kruskal-Wallis 1-way ANOVA test followed by the Dunn’s test corrected for multiple comparisons. For the qVOA experiments, IUPM with and without galunisertib were compared in the different LRA conditions using 2-way ANOVA followed by the Holm-Šídák multiple comparisons test. Before and after galunisertib SUV, tissue VL data as well as anti–SIV-env titers and frequency of cells producing different cytokine combinations following peptide pool stimulation were compared using a mixed-effect model with the Holm-Šídák multiple comparisons test, with a single pooled variant. DC phenotype between TGF-β–DCs and moDCs was compared using the ratio paired 2-tailed *t* test. Statistical analysis was performed using Prism GraphPad 9.3 and R. *P* < 0.05 was considered statistically significant.

### Study approval.

All animal experiments were conducted following guidelines established by the Animal Welfare Act and the NIH for housing and care of laboratory animals and performed in accordance with institutional regulations after review and approval by the Institutional Animal Care and Usage Committees of the University of Louisiana at Lafayette (2016-8761-067; protocols 8761-1709 and 8761-1802). Human PBMCs and CD4^+^ T cells were from deidentified donors and exempt from IRB approval.

## Author contributions

SS, MSA, JMA, IF, MDM, CTT, MRA, VBH, and EM performed assays and analyzed the data; YT and PJS analyzed the PET/CT images; BO and AG performed the galunisertib PK analysis; MA, C Carter, LM Shirreff, DB, and FJV coordinated the macaque studies and performed PET/CT imaging; LM Simons, JFH, and RLR performed the viral sequencing; RLR analyzed the data; JA, C Cicala, and IS contributed to the ex vivo studies, data interpretation, and drafting of the manuscript; TJH, FJV, and EM interpreted the data; and EM conceptualized the studies and wrote the manuscript.

## Supplementary Material

Supplemental data

Supplemental video 1

Supplemental video 2

Supplemental video 3

Supplemental video 4

Supplemental video 5

Supplemental video 6

Supplemental video 7

## Figures and Tables

**Table 1:**
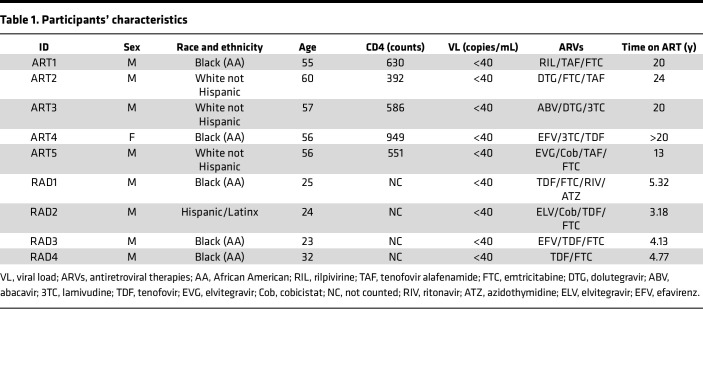
Participants’ characteristics

**Table 2:**
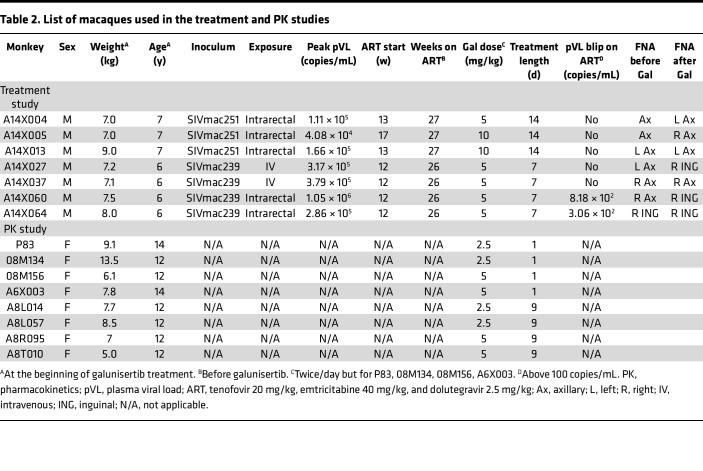
List of macaques used in the treatment and PK studies

## Version 1 09/20/2022

In-Press Preview

## Version 2 11/08/2022

Electronic publication
