# Supplementary material for: Blockade of TGF-β signaling reactivates HIV-1/SIV reservoirs and immune responses in vivo
Source: JCI Insight. 2022 Nov 8;7(21):e162290. doi: 10.1172/jci.insight.162290 (PMC9675457; doi:10.1172/jci.insight.162290)
Supplement: Supplemental data [file jciinsight-7-162290-s056.pdf]

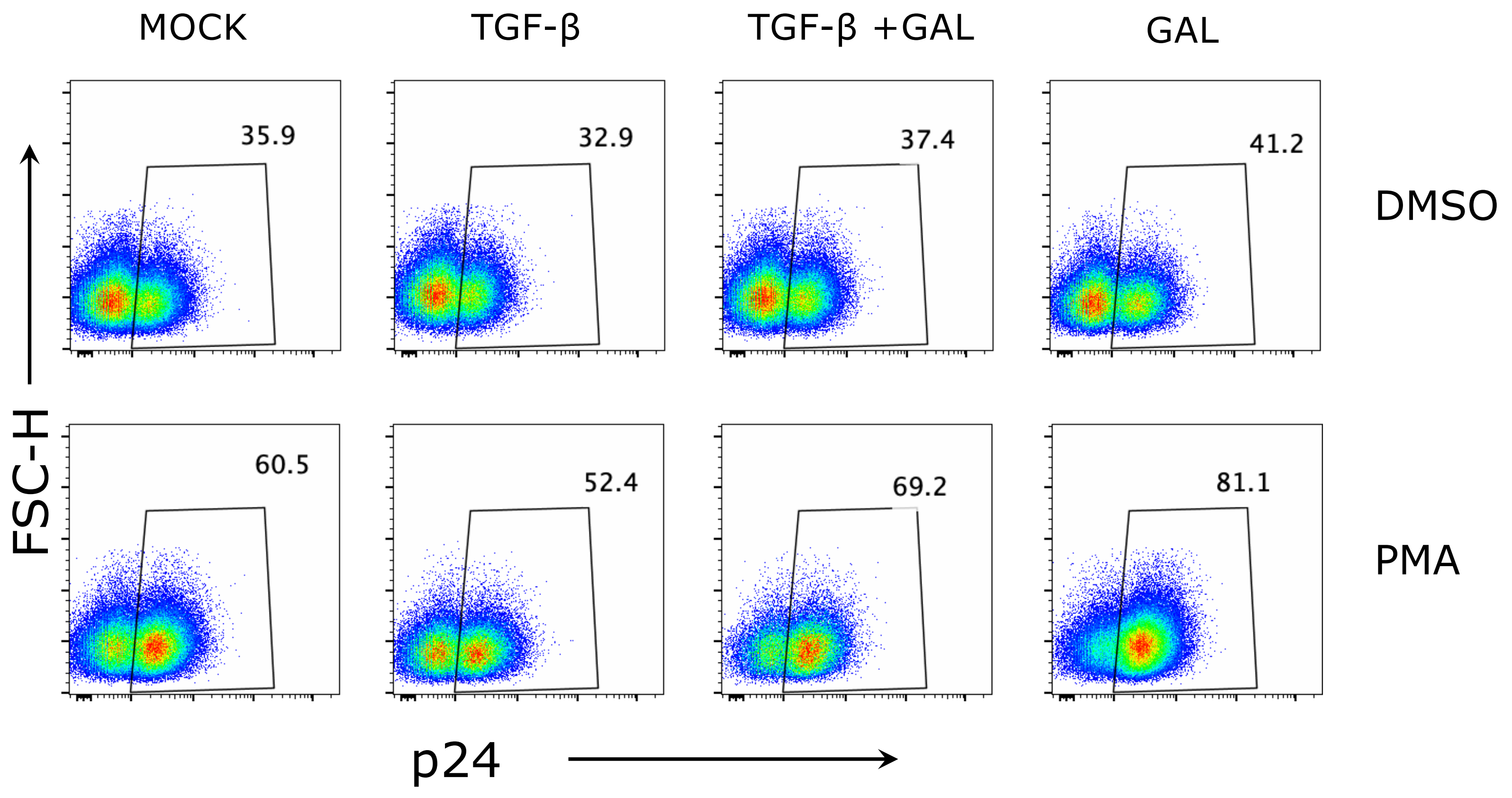

Figure S1. TGF-β inhibits HIV-1 latency reactivation by PMA in ACH-2 cells. Flow cytometry multicolor plots of p24 ICS from one representative experiment with ACH-2 cells treated with TGF-β1 (10ng/ml) or galunisertib (GAL; 1μM) or both overnight.

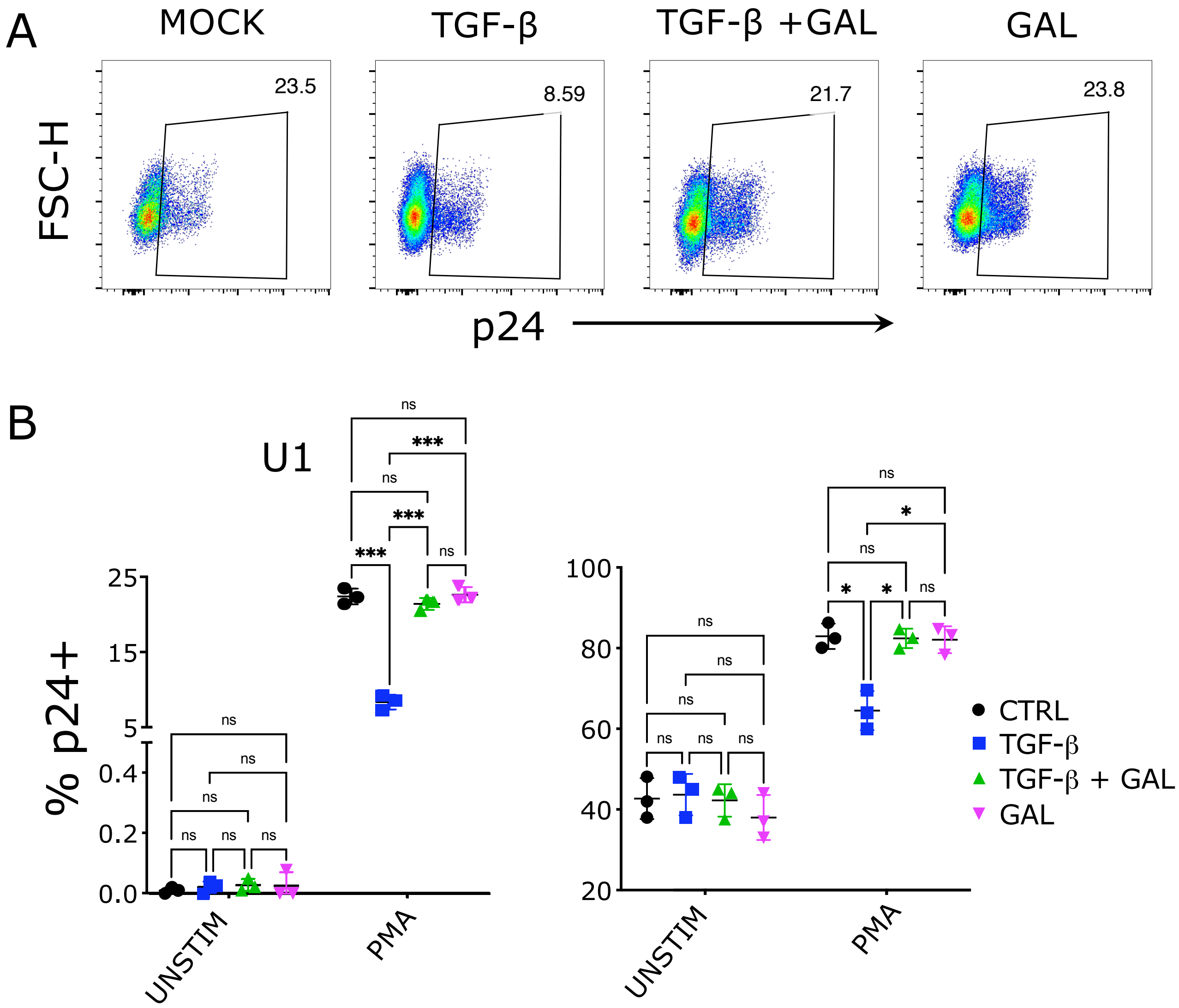

Figure S2. More profound effect of TGF-β in absence of FBS. A) Flow cytometry multicolor plots of p24 ICS from one representative replicate of U1 cells treated with PMA in X-Vivo 15 media without FBS and TGF-β1 (10ng/ml) or galunisertib (GAL; 1μM) or both overnight. **B-C) E**xperiments in triplicate with U1 (B) or ACH-2 (C) cells cultured in X-Vivo 15 media without FBS and treated with TGF-β1 (10ng/ml) or galunisertib (GAL; 1μM) or both overnight. Brwon-Forsythe and Welch Anova with Dunnett's T3 correction for multiple comparisons was used to compare the different treatments. (Significant *p*-values of α<0.05 (*), α<0.01 (**) and α <0.001 (***) are indicated)

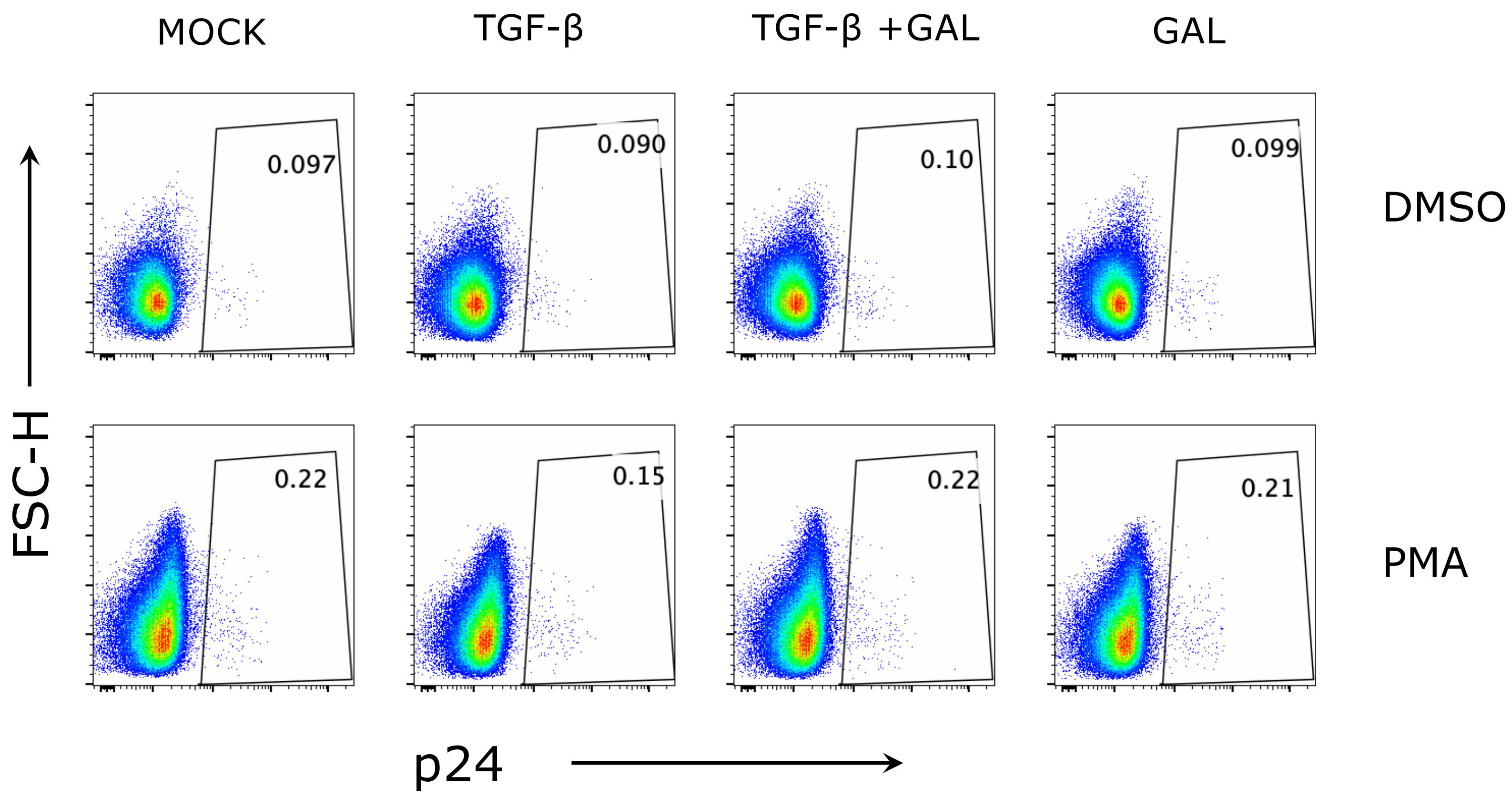

Figure S3. TGF-β inhibits HIV-1 latency reactivation by PMA in primary CD4⁺ T cells. Flow cytometry multicolor plots of p24 ICS from one representative experiment of primary CD4⁺ T cells model of HIV-1 latency. Freshly isolated CD4⁺ T cells were activated and infected with HIV-ADA by spinoculation. After 48hrs, cells were stimulated with PMA in presence vs absence of TGF-β1 (10ng/ml) or galunisertib (GAL; 1μM) or both overnight.

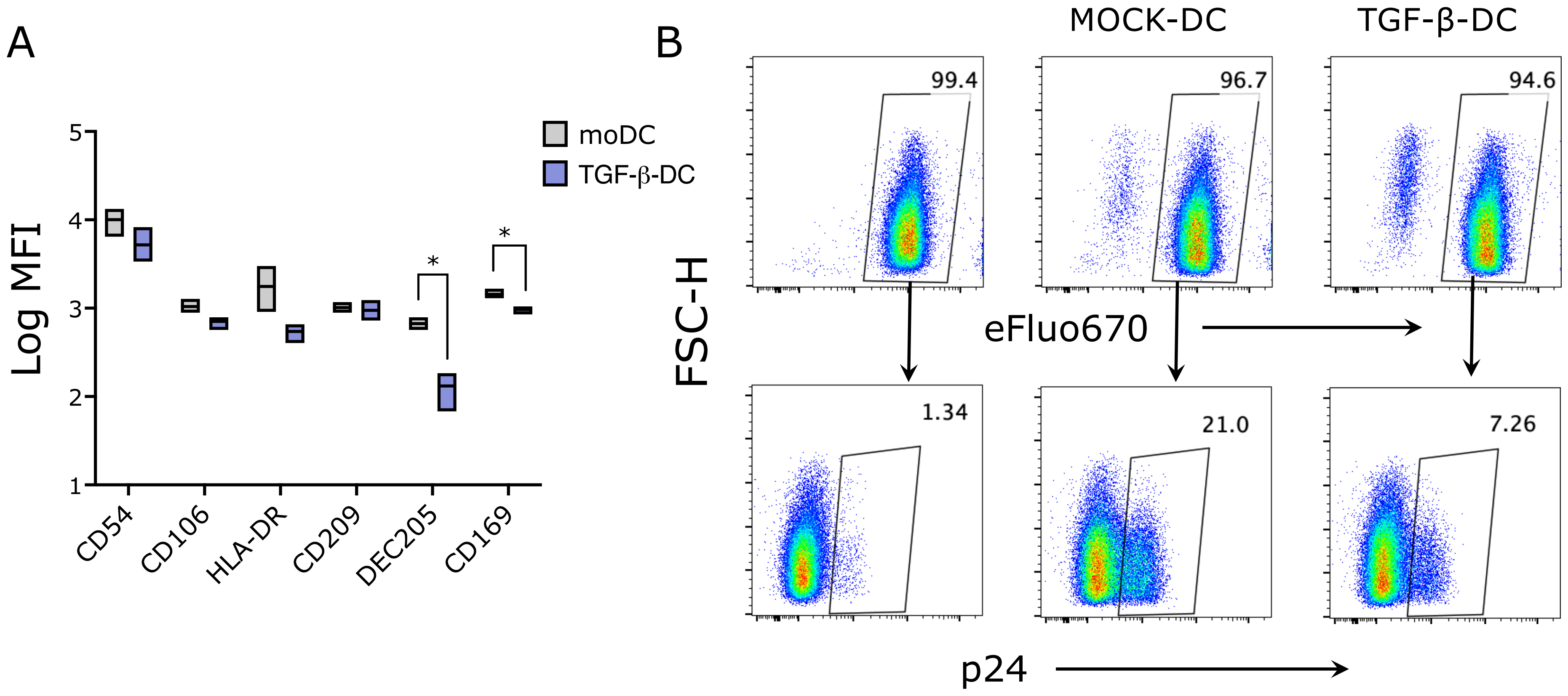

Figure S4. TGF-β-moDCs reactivate latent HIV-1 less than untreated moDCs. A) High-low box plots (line at median) of Log MFI of expression of the indicated marker on moDCs or TGF-β-moDCs from 3 different donors are shown. Paired t-test was used to compare moDCs and TGF-β-moDCs (Significant *p*-value of $\alpha<0.05$ (*) is indicated) . B) One representative example of multicolor plots from a p24 ICS of eFluo670-labelled U1 cells co-cultured with moDCs or TGF-β-moDCs overnight.

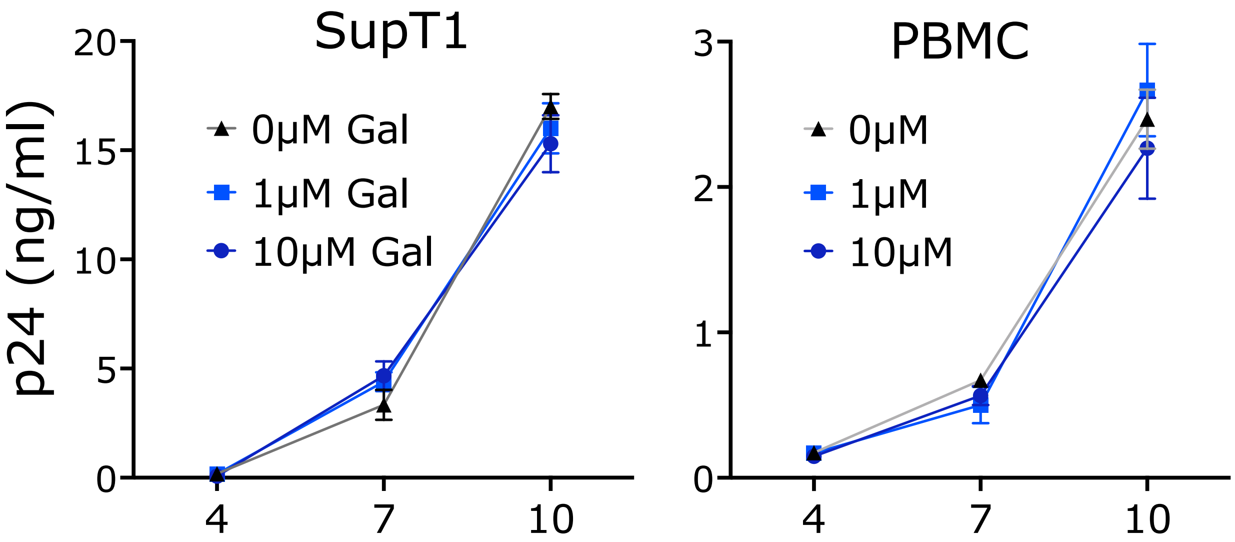

Figure S5. Galunisertib does not impact *in vitro* HIV-1 replication. The effect of galunisertib on HIV replication was tested in SupT1-M10 and PBMC from HIV- donors infected at low MOI to detect an increase in replication. One of 2 experiments in triplicates in SupT1 (left) and PHA-PBMC (right) infected with HIV Bal at MOI of 0.01 ±Gal (added every 2 days).

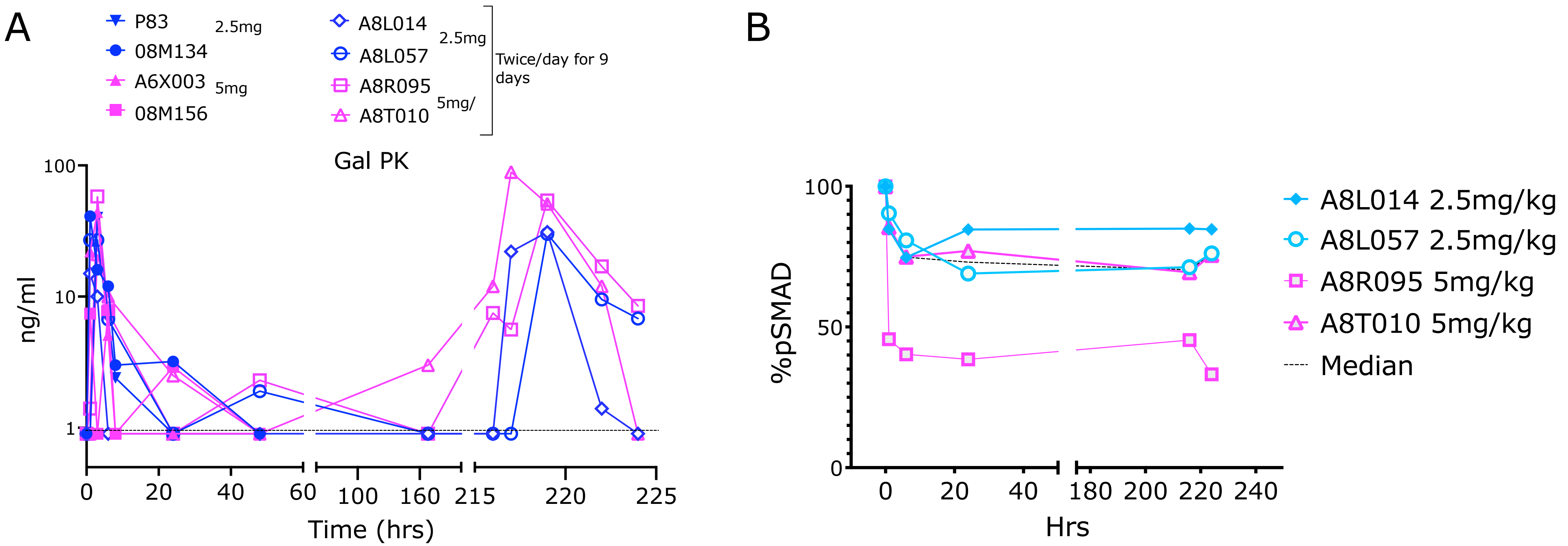

Figure S6. Galunisertib PK and PD in rhesus macaques. A) 8 naïve rhesus macaques were treated with galunisertib only once (n=4) or twice per day for 9 days and plasma collected at the indicated time (hrs) after the first dose of galunisertib. Galunisertib concentrations in plasma samples were measured by LCMS. B) PBMC from 4 animals treated for 9 days with galunisertib at 2.5mg/kg or 5mg/kg twice daily were anayzed for phosphoSMAD2/3 amounts and total pSMAD2/3. The percentage ration of Phospho over Total- SMAD2/3 is shown at the indicated time point after th efirst galunisrtib treatment.

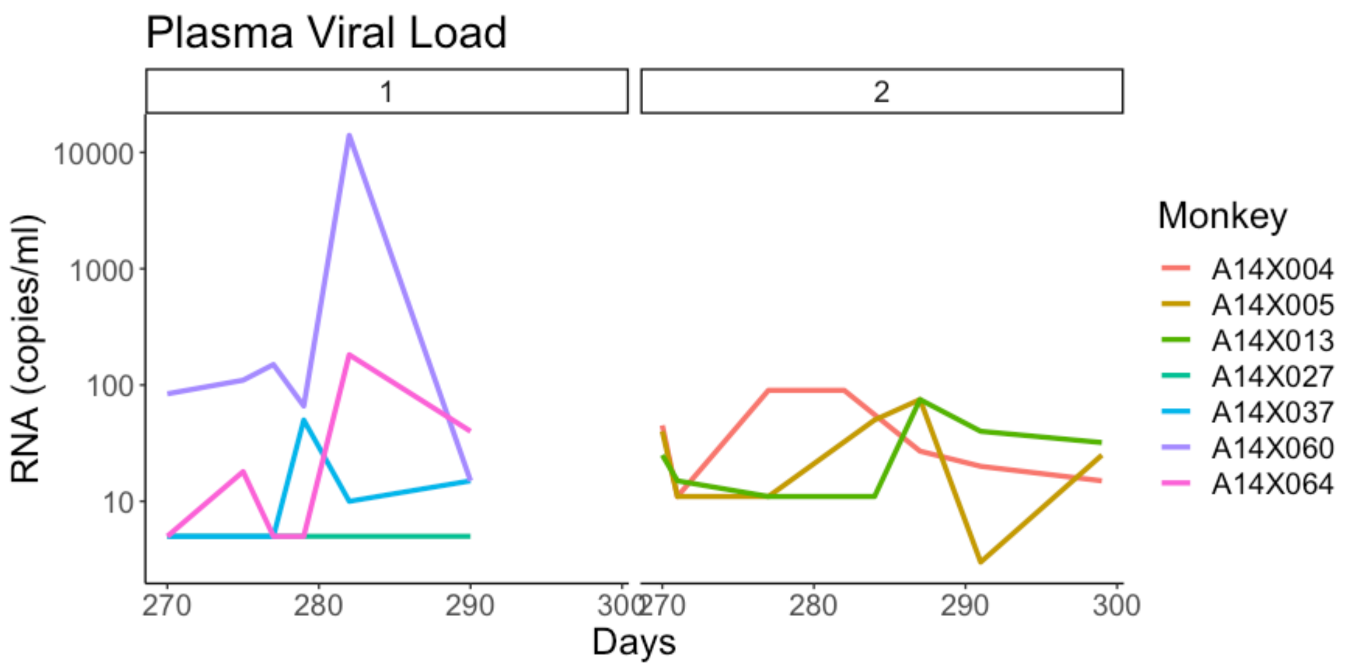

Figure S7. Plasma VL during galunisertib treatment as detected by Leidos. Plasma samples collected before, during and after the 7 or 14 days treatment with galunisertib were assayed for plasma viral loads at Leidos with an ultrasenstitive (LLOD 3 to 5copies/ml) assay and the results shown here. Pilot 1 indicates the study with 4 animals that were treated for 7 days. Pilot 2 indicates the study with 3 animals that were treated for 14 days.

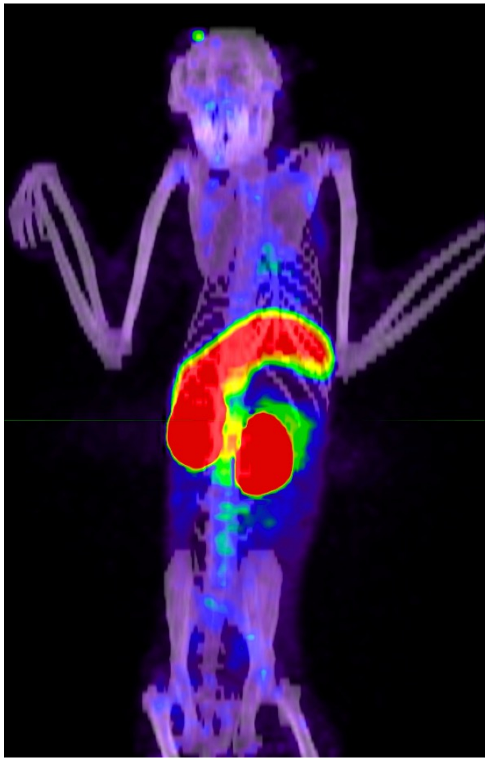

Figure S8 A control IgG1 probe accumulates in liver and kidneys with no binding in tissues. A ^{64}Cu-DOTA-F(ab')$_2$ pIgG1 control Fab probe was generated by digestion of the Fc portion of a rhesus isotype control IgG1 obtained from the NHP reagent repository. MIPs were generated using the MIM software and set to a numerical scale of 0-1.5 SUVbw visualized with the color scale *PET Rainbow* (same settings used for the galunisertib treated animals). The PET signal accumulates as expected in the liver, kidney and spleen.

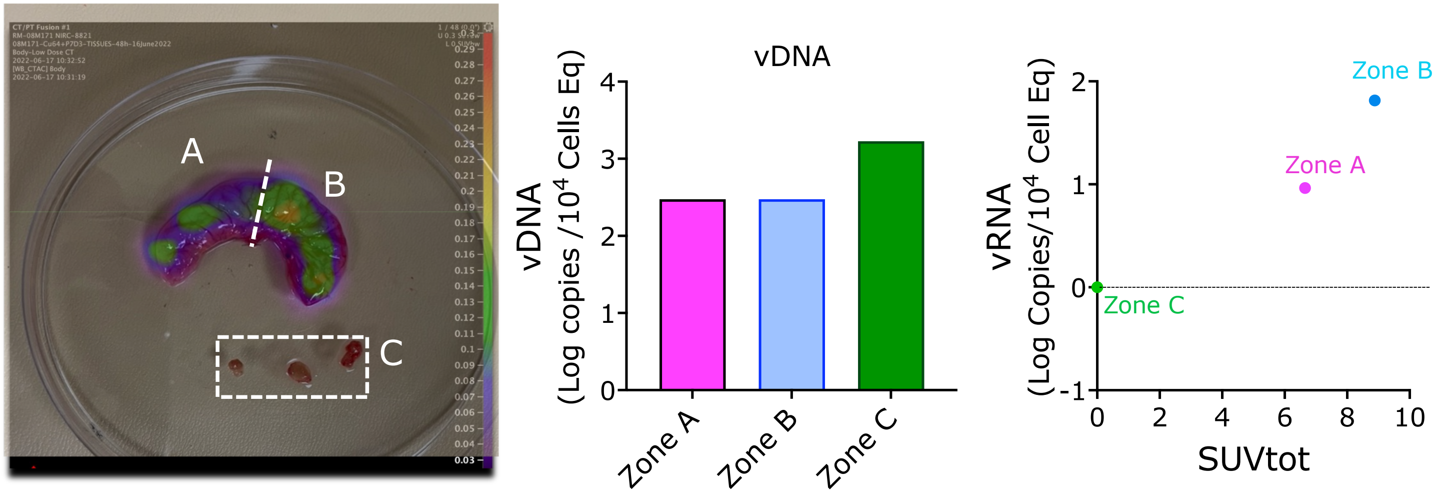

Figure S9. PET SUVtot in a specific tissue area correlated with CA-vRNA levels. A piece of small intestine was collected during a major resection surgery after being identified as positive during a whole-body PET/CT scan of an SIV infected macaque (08M171). Adjacent MLN were also collected. **A)** The tissue and MLN were placed on a petri dish and a picture taken before re-scan. The picture of the tissue on the petri dish was overlapped to the PET image and the tissue sectioned in 2 based on the level of PET signal along the white dotted line as shown. Each piece was snap frozen and tested for CA-vDNA/RNA by qPCR. **B)** The levels of CA-vDNA in each tissue area are shown **C)** The correlation between SUVtot in each tissue area and CA-vRNA levels are shown.

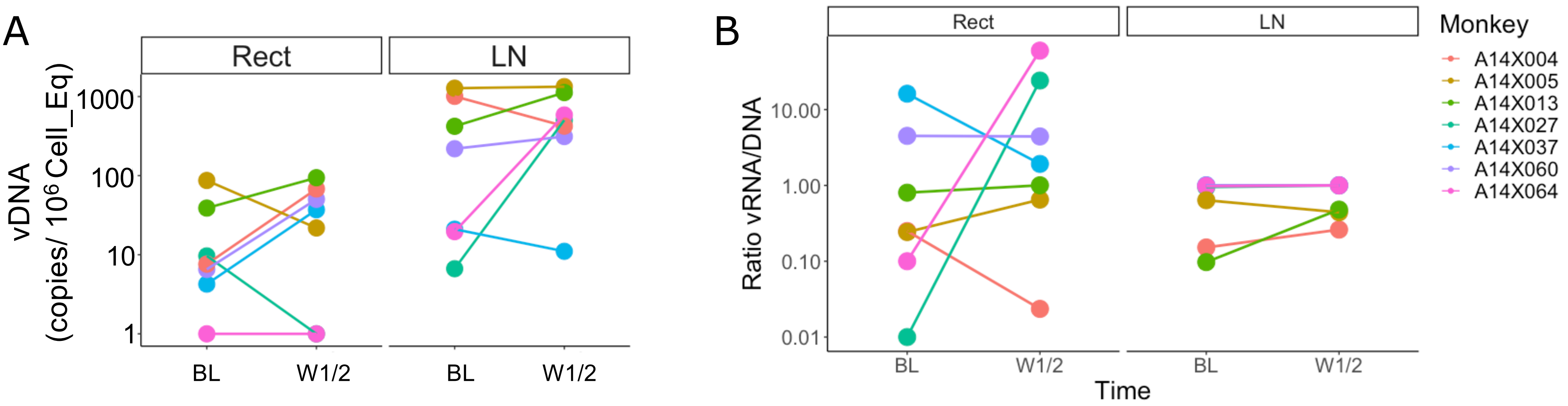

Figure S10. Ratio vRNA/DNA in colorectal and lymph node biopsies. A) Copies of SIV-gag DNA per cell equivalent are shown for colorectal biopsies and lymph nodes (FNA) collected before and after galunisertib treatment. B) The ratio of vRNA copies/ vDNAcopies for each tissue is shown. In the LNs the ratio is 1 and unchanged for 4 of the 7 animals and the lines overlap each other. Differences between BL an W1/2 are not significant by Wilcoxon matched-pairs test.

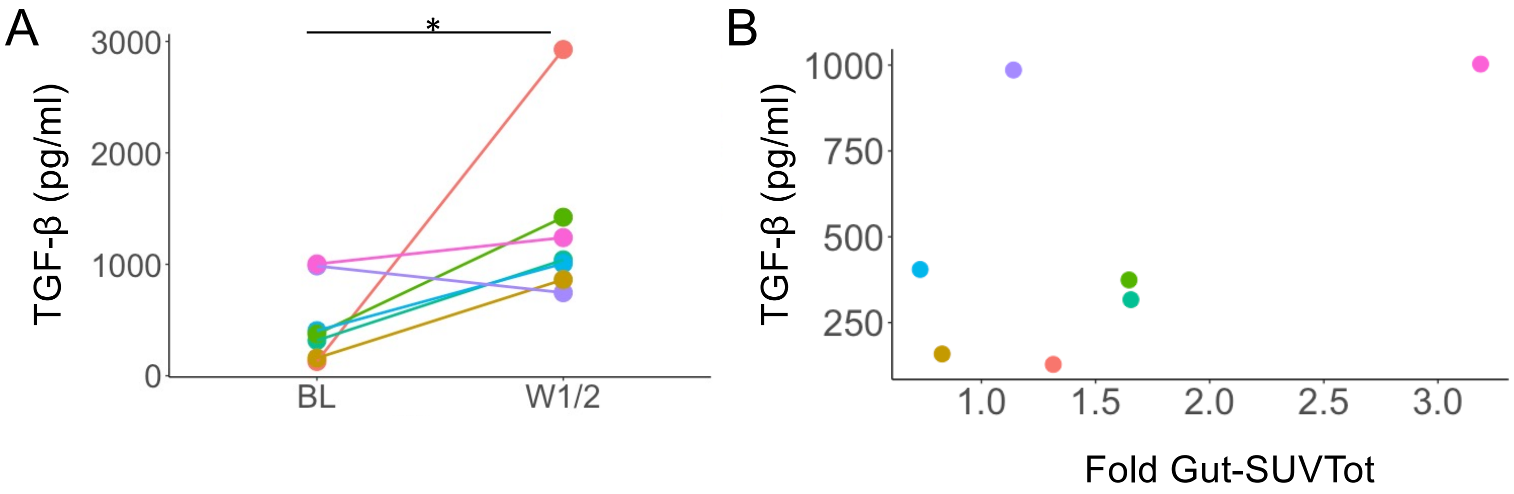

Figure S11. TGF-β levels do not predict the effect of Gal on the viral reservoir. A) The concentration of total TGF-β in the plasma before and after galunisertib as detected by ELISA is shown. Wilcoxon matched-pairs test was used to compare BL with W1/2 data, Significant *p*-value of α<0.05 (*) is shown. B) No correlation is present between the concentration of TGF-β before treatment in blood and the change in Gut-SUVTot in each animal after galunisertib treatment (p=0.199; Person R= 0.304)

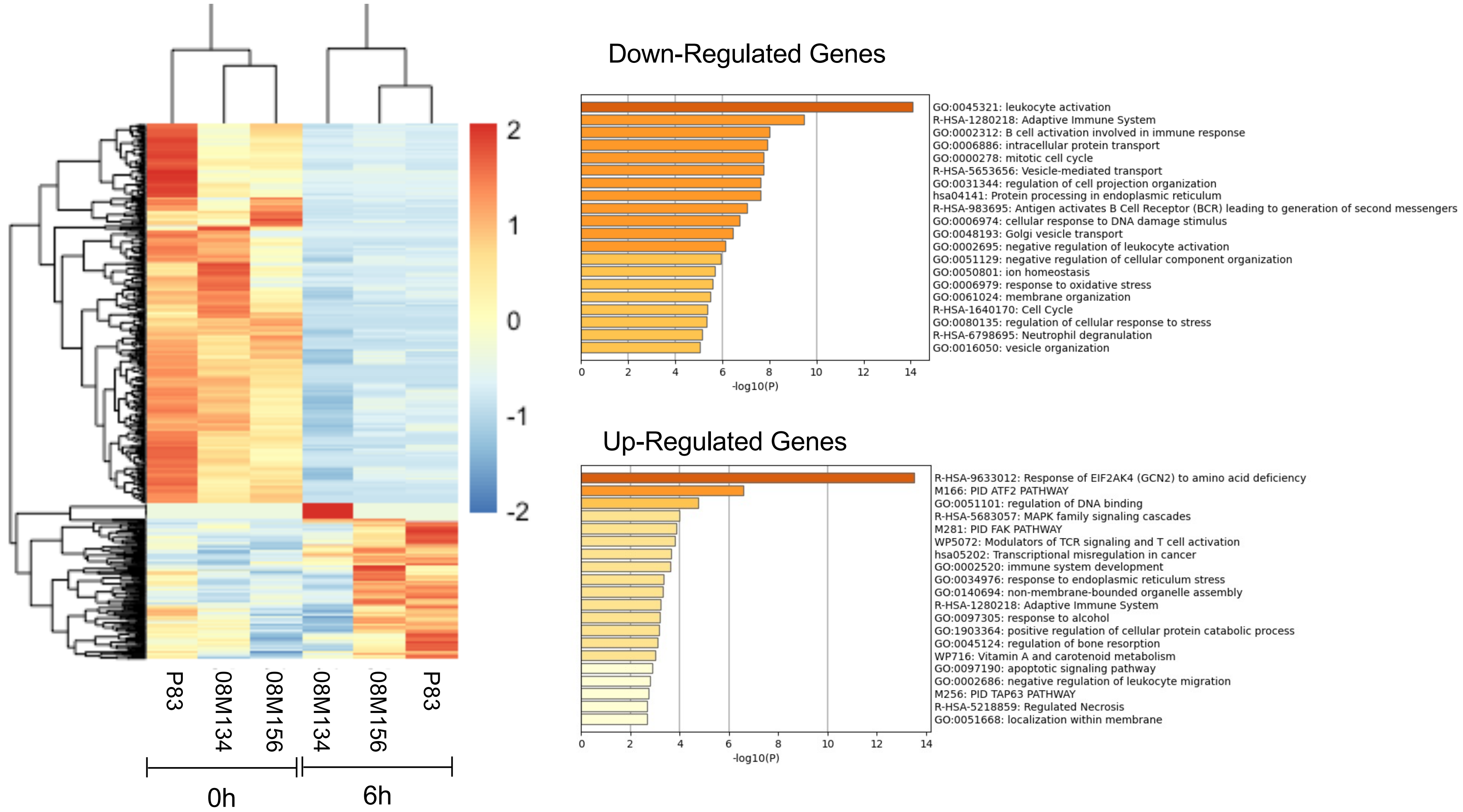

Figure S12. Galunisertib treatment stimulates immune cell pathways. CD4+ T cells were isolated from PBMC of naïve macaques before and after (6hrs) treatment with galunisertib for PK studies (see Fig S5) and TotalRNAseq was performed at the Northwestern University Center for Genetic Medicine NUSeq core. Data were analyzed

Supplemental Table 1. List of antibodies used for moDC phenotyping and PBMC ICS.

**Marker**	**Dye**	**Clone**	**Manufacturer**
HLA-DR	BV605	L243	BioLegend
CD54	AF700	HA58	BioLegend
CD106	PE	STA	BioLegend
CD209	BV421	9E9A8	BioLegend
CD169	PEcy7	7239	BioLegend
DEC205	AF647	MMRI7	BioLegend
CD3	V450	SP34-2	BD Biosciences
CD4	BUV395	L200	BD Biosciences
CD8	PCP-Cy5.5	SK1	BD Biosciences
IFN-$_{\gamma}$	Alexa Fluor 700	B27	BD Biosciences
TNF-α	FITC	MAb11	BioLegend
IL-2	BV605	MQ1-17H12	BioLegend

Movie Legends

Movies 1-7. Timeline maximum intensity projections (MIP) of fused PET and CT scans for macaques A14X027 (Movie 1), A14X037 (Movie 2), A14X060 (Movie 3), A14X064 (Movie 4) before and after 1-week of galunisertib treatment and for macaques A14X004 (Movie 5), A14X005 (Movie 6) and A14X013 (Movie 7) before, after 1-week and after 2-weeks of galunisertib treatment. Fusions of the whole-body CT and PET scans were generated for each timepoint. MIPs were generated using the MIM software and set to a numerical scale of 0-1.5 SUVbw (0-0.3 SUVbw after subtraction of liver and kidney signal for Movie 5) visualized with the color scale *PET Rainbow*. The MIP were scaled to the same physical size using the dimensions to the scanner's visible range, and the scanner bed was removed from the CT scan. The MIP are displayed in chronological order, qualitatively demonstrating the changes in intensity and location of PET signals. Each movie generated in MIM as a series of MIP for one full rotation (~7 seconds) was captured using QuickTime.

Movie S1 A14X004 with liver and kidney signal. Timeline maximum intensity projections (MIP) of fused PET and CT scans for macaque A14X004 including the liver and kidney signal at a scale of 0-1.5 SUVbw.

Movie S2 PET/CT images of an SIV infected macaque injected with a control probe. Timeline maximum intensity projections (MIP) of fused PET and CT scans for macaque A8L057 infused with a 64Cu-DOTA-F(ab')2 IgG1 isotype control at a scale of 0-1.5 SUVbw.
